# Differentially Expressed Genes and Molecular Pathways in an Autochthonous Mouse Prostate Cancer Model

**DOI:** 10.3389/fgene.2019.00235

**Published:** 2019-03-26

**Authors:** Shiv Verma, Sanjeev Shukla, Mitali Pandey, Gregory T. MacLennan, Sanjay Gupta

**Affiliations:** ^1^Department of Urology, School of Medicine, Case Western Reserve University, Cleveland, OH, United States; ^2^The Urology Institute, University Hospitals Cleveland Medical Center, Cleveland, OH, United States; ^3^Vancouver Prostate Center, Vancouver, BC, Canada; ^4^Department of Pathology, University Hospitals Cleveland Medical Center, Cleveland, OH, United States; ^5^Department of Nutrition, Case Western Reserve University, Cleveland, OH, United States; ^6^Department of Urology, Louis Stokes Cleveland Veterans Affairs Medical Center, Cleveland, OH, United States; ^7^Division of General Medical Sciences, Case Comprehensive Cancer Center, Cleveland, OH, United States

**Keywords:** biomarkers, therapeutic target, prostate cancer, gene expression, TRAMP mice

## Abstract

Prostate cancer remains a major public health problem and the second leading cause of cancer-related deaths in men in the United States. The present study aims to understand the molecular pathway(s) of prostate cancer which is essential for early detection and treatment. Dorsolateral prostate from 20 week transgenic adenocarcinoma of the mouse prostate (TRAMP) mice, which spontaneously develops prostate cancer and recapitulates human disease and age-matched non-transgenic littermates were utilized for microarray analysis. Mouse genome network and pathway analyses were mapped to the human genome using the Ingenuity Pathway Analysis (IPA) database for annotation, visualization, and integrated discovery. In total, 136 differentially expressed genes, including 32 downregulated genes and 104 upregulated genes were identified in the dorsolateral prostate of TRAMP, compared to non-transgenic mice. A subset of differentially expressed genes were validated by qRT-PCR. Alignment with human genome database identified 18 different classes of proteins, among these, 36% were connected to the nucleic acid binding, including ribosomal proteins, which play important role in protein synthesis—the most enriched pathway in the development of prostate cancer. Furthermore, the results suggest deregulation of signaling molecules (9%) and enzyme modulators (8%) affect various pathways. An imbalance in other protein classes, including transporter proteins (7%), hydrolases (6%), oxidoreductases, and cytoskeleton proteins (5%), contribute to cancer progression. Our study evaluated the underlying pathways and its connection to human prostate cancer, which may further help assess the risk of disease development and progression and identify potential targets for therapeutic intervention.

## Introduction

Prostate cancer is the second leading cause of cancer-related deaths in men in the United States (Cronin et al., 2018; Negoita et al., 2018). According to an estimate by the American Cancer Society, ~ 164,690 new cases of prostate cancer were diagnosed and 29,430 deaths occurred from the disease in the year 2018 (www.cancer.org). Prostate cancer is characterized by the accumulation of epigenetic and genetic events (Gasi Tandefelt et al., 2014) and is majorly affected by diet and lifestyle factors (Nelson et al., 2007; Barbieri and Tomlins, 2014; Discacciati and Wolk, 2014; Gandhi et al., 2018) Despite the existence of screening and preventive strategies, prostate cancer remains a major public health problem. Thus, understanding the molecular pathways of prostate cancer is essential for early detection and treatment.

Elucidation of molecular pathways leading to prostate cancer development and progression is difficult to attain in patients, as these studies may require decades of observation. Such studies are also hindered by the need for obtaining repeat biopsies from the patients under surveillance. Consequently, it is advantageous to evaluate molecular pathways of prostate cancer development and progression in animal models that recapitulate human disease. To date, numerous mouse models are developed that have advanced the area of prostate cancer research. Some established animal models include tumor suppressor knockout PTEN mouse (Wang et al., 2003); gain of function model such as the c-myc mouse (Ellwood-Yen et al., 2003); and various conditional knockouts of multiple genes including the NKX3.1 (Kim et al., 2002); p27/Kip1 knockout (Gao et al., 2004) and the p53 and retinoblastoma (Rb) double knockout mouse (Zhou et al., 2006), which correlate with possible similar alterations in human prostate cancer.

The transgenic adenocarcinoma of the mouse prostate (TRAMP) has been established as an excellent mouse model of prostate cancer (Greenberg et al., 1995; Gingrich et al., 1996). Male TRAMP mouse develops progressive, multifocal and heterogeneous disease, which emulates histological and molecular features of human prostate cancer (Kaplan-Lefko et al., 2003). TRAMP mice express the large T-antigen from Simian Virus 40 downstream of the minimal −426 to +28 bp regulatory element of the androgen-responsive rat *probasin* (Pb-Tag) gene promoter. Upon sexual maturity (~12 weeks) male hemizygous TRAMP mice abrogate tumor suppressor activity of p53 and Rb proteins through the simian virus 40 Tag. The loss of functional p53 and Rb predisposes epithelial cells to increase survival and proliferation signals, leading to molecular abnormalities. Initial development and progression of prostate cancer in TRAMP mice is androgen-dependent and is exceedingly reproducible. TRAMP mice exhibit low-grade prostatic intraepithelial neoplasia (PIN) by 6 weeks, progressing to high-grade (HG)-PIN by 12 weeks. Focal adenocarcinoma is observed between 12 and 18 weeks, with subsequent progression to moderate/poorly differentiated carcinoma within 24 weeks. Hundred percent of TRAMP mice, by 28 weeks, harbor metastatic prostate cancer in liver, lymph nodes, lungs, and occasionally in bone without any treatment (Kaplan-Lefko et al., 2003; Berman-Booty et al., 2012). TRAMP mice has been extensively studied to understand the genetic and epigenetic alteration, elucidating the role of various genes and pathways relevant for human prostate cancer development and progression (Kaplan-Lefko et al., 2003; Shukla et al., 2007, 2013; Berman-Booty et al., 2012) We and others have elucidated some of the known pathways that include the PI3K-Akt, Androgen Receptor, NF-κB, FOXO3A, IGF-growth axis, Wnt signaling pathways perturbed in the prostate of TRAMP mice (Shukla et al., 2004, 2007, 2009, 2016; Liao et al., 2005; Yang et al., 2006). In previous years, microarray studies assessing the transcriptome and proteome were performed and the data was associated with prostate cancer (Morgenbesser et al., 2007; Haram et al., 2008; Zhang et al., 2011). A microarray study performed on TRAMP mouse prostate mimicking advance-stage disease revealed a subset of genes with concordant expression in mouse and human prostate cancer having marked difference in the expression of genes regulating metastases and cell cycle progression (Haram et al., 2008). Simultaneously proteome changes have been demonstrated in lobe-specific manner in TRAMP mice connecting to different pathway networks (Zhang et al., 2011). However, functional dependency among pathways has not been elucidated.

In the present study, gene expression profiling was assessed using Affymetrix (Santa Clara, CA) Gene Chip Mouse Expression Set 430 array representing >34,000 genes in the dorsolateral prostate of 20 week old TRAMP mice and age-matched non-transgenic littermates. Identification of differentially expressed genes (DEGs), gene ontology (GO) and Ingenuity Pathway analysis (IPA) analyses were performed on mouse and human genomes. Protein-protein interaction (PPI) networks and sub-networks were also constructed and overlapped with cancer to identify key target genes and major pathways involved in human prostate cancer. Using the aforementioned bioinformatics methods, modification of gene expression in prostate cancer was analyzed by identifying significant DEGs and pathways, which may provide a novel insight in identifying potential targets for prevention and/or treatment of prostate cancer.

## Materials and Methods

### Animals

Male and female heterozygous C57BL/TGN TRAMP mice, Line PB Tag 8247NG were purchased as breeding pairs from The Jackson Laboratory (Ann Arbor, MI). The animals were bred and maintained at the AAALAC-accredited Animal Resource Facility of Case Western Reserve University. Housing and care of animals was in accordance with the guidelines established by the University's Animal Research Committee and with the NIH Guidelines for the Care and Use of Laboratory Animals. Transgenic males for these studies were routinely obtained as [TRAMP x C57BL/6]F1 or as [TRAMP x C57BL/6]F2 offspring. Identity of transgenic mice was established by PCR-based DNA screening as previously described (Gupta et al., 2000). All experimental protocols were approved by the Case Western Reserve University Institutional Animal Care and Use Committee (IACUC #2013-0093).

### Tissue Processing

Twenty week old TRAMP mice and non-transgenic littermates were euthanized, tissues were harvested in an RNase-free manner. Incisions were made in the anterior abdomen with RNase-Zap (Ambion, Austin, TX) treated forceps and scissors. The vas deferens, ureters, and any connective tissue were clipped away from the genitourinary (GU) complex. The entire GU complex consisting of the bladder, seminal vesicles and the prostate along with the urethra was removed from the abdominal cavity and placed in cold RNase-free PBS (Ambion) in an RNase-Zap treated petri-dish and micro-dissected to excise the dorsolateral prostate, either snap frozen in liquid nitrogen and stored at −80°C until RNA isolation or used immediately for RNA isolation. Small sections of the prostates from TRAMP mice and non-transgenic littermates were preserved in buffered formalin. Samples were stained with hematoxylin and eosin for histological analysis.

### Histology of TRAMP Mouse Prostate

Histological sections of the prostate were reviewed by light microscopy for the presence/absence of prostate cancer classified as PIN (epithelial stratification with occasional mitotic figures or cribriform pattern); well-differentiated (multiple epithelial mitotic figures and apoptotic bodies, invasive glands with stromal hypercellularity), moderately-differentiated (many acini completely filled with tumor yet still forming some glandular structures), or poorly-differentiated (sheets of malignant cells with little or no glandular formation) prostate cancer; or atrophic glands only (no identifiable tumor deposits) according to the grading system previously described for TRAMP mice (Kaplan-Lefko et al., 2003; Berman-Booty et al., 2012).

### RNA Isolation

Total RNA was extracted from frozen mouse prostate tissue specimen isolated by TRIzol (Invitrogen Corporation). Briefly, tissue was placed in cold TRIzol (4°C) and immediately homogenized using a TissueLyser (Qiagen, Valencia, CA). The protocol for TRIzol isolation was then followed as per vendor's instructions. The quantity of RNA was assessed by NanoDrop ND-1000 Spectrophotometer (NanoDrop, Wilmington, DE). RNA quality control (QC) was evaluated with Agilent's RNA 2100 Bio-analyzer with 28S/18S ratio and RIN determined by 2100 Expert Software (Santa Clara, CA). The final RNA samples selected passed the RIN number 7 or above and dorsolateral prostates from 3 non-transgenic and 4 TRAMP mice were used as biological and technical replicates for microarray experiment.

### Microarray and Data Analysis

For microarray experiment, 250 ng of total RNA was amplified using Ambion's Message Amp II aRNA Amplification Kit. Biotin-UTP was incorporated during the overnight *in vitro* transcription step as per the manufacturer's protocol. Gene expression was assessed using Affymetrix's Gene-Chip Mouse Genome 430 arrays (Chip# 4059997) representing >34,000 mouse genes. 15 μg of cRNA was fragmented and hybridized to arrays according to the manufacturer's protocol. Arrays were scanned with the GeneChip Scanner 3000 and GeneChip Operating Software acquired raw fluorescence signal. Scanned array images were analyzed by dChip as previously described (Haram et al., 2008). The TRAMP microarray dataset was submitted to public repository GEO with accession number GSE119205.

### Gene Ontology Analysis

Gene ontology (GO) analysis was conducted on differentially expressed transcripts in TRAMP mice compared with non-transgenic littermates using the PANTHER (Protein Analysis through evolutionary relationships) database which is a part of GO designed to classify proteins, and their genes for high-throughput analysis to access relational database for functional annotations. The PANTHER database was used to identify either protein classes or GO annotations overrepresented in our data compared to a reference mouse and human genome database separately. *P*-values were adjusted using a Bonferroni correction.

### Ingenuity Pathway Analysis (IPA)

The microarray dataset which includes log2 fold changes and differential regulation status for each identified transcript was uploaded into Ingenuity Pathway Analysis (IPA) using the core analysis platform (Qiagen, Ingenuity Systems, Redwood City, CA). The differentially expressed microarray data of TRAMP mice was matched with those in Ingenuity knowledge human database and also with IPA mouse database separately. A comparison spreadsheet was constructed on the basis of sequence identity. Molecular networks were created, the data divided into biological functions that were overrepresented in the dataset, and overrepresented canonical pathways; unmapped RNA were excluded from further analysis. To evaluate the definite overrepresented pathway(s), or to remove the chances of any randomness in data with reference to *p*-value, another statistical Benjamin-Hochberg (B-H) parameter was used. The results described are based on B-H test data.

### Overlapping Pathways and Network Analysis

Interactive networks, pathways and functions analysis was performed on this list of genes using commercial system biology oriented package Ingenuity Pathways Analysis (IPA 5.0) (www.ingenuity.com). Node color indicates upregulated genes (red) and downregulated genes (green). Edge line and arrow between nodes represents direct (solid line) and indirect (dashed lines) interactions between molecules as supported by IPA knowledge database including annotations, synonyms and over millions of published biological interactions. In case of interactive networks, all the identified genes were mapped to genetic networks available in the ingenuity database and were ranked by their individual score. The activation score was analyzed with IPA software, the signaling pathways which are activated have a Z score ≥ 2, and those which are inhibited scored ≤ −2, respectively. The primary purpose of the activation z-score is to infer the activation states of predicted transcriptional regulators.

### Quantitative Real-Time qRT-PCR Validation

To validate the microarray results, real-time PCR was performed to investigate the differential expression pattern for a subset of selected genes including internal control. Oligo's (Integrated DNA Technologies) were designed using Primer3 software (Whitehead Institute of Biomedical Research MIT, Boston, MA) (Supplemental Table 1). All reactions were performed in triplicate (three biological and three technical replicates) along with no template controls. The genes selected for validation are: ATP synthase 1 (*ATP15L*), ADP-ribosylation factor GTPase activating protein 2 (*ARFGAP2*), dimethyladenosine transferase 1-like (*DIMT1*), germ cell-less homolog L1 (*GMCL1*), kruppel-like factor 4 (*Klf4*), mitochondrial ribosomal protein L23 (*Mrpl23*), myeloperoxidase (*Mpo*), nascent polypeptide-associated complex alpha polypeptide (*Naca*), ribosomal protein L22 (*RPL22*), sclerostin domain containing 1 (*Sostdc1*), small proline-rich protein 1B (*SPRR1a*), Sp140 nuclear body protein (*SP140*), transglutaminase 4 (*TgM4*), and voltage-dependent anion channel 3 (*VDAC3*). Primer design for the genes are shown in Supplemental Table 1. The relative expression of above genes were compared with the expression of endogenous genes; *GAPDH* (NM_008084), and *Actin* (NM_007393) as internal control in the reaction. Five microliters of each 10 μg/ml sample were added for a total 25 μl volume with SYBR green (Quanta, USA). The thermal cycler program used started at 48°C for 30 min to generate cDNA. The program then proceeded with 95°C for 15 min for initial denaturing, followed by 40 cycles of 95°C for 15 sec, 60°C for 40 sec, and 72°C for 35 s to collect cycle threshold (Ct) values. Following PCR, the samples then processed through the dissociation thermal profile on the ABI Prism 7500 Sequence detection system (Applied Biosystems) to obtain a dissociation curve for each sample. Expression values of each gene were normalized to the expression of *GAPDH* and *Actin* of a given sample. From multiple reference gene the geometric mean (GM) was calculated and used to evaluate the expression ratio and normalize the data. The data was randomized with itineration value of 2000. The 2-ΔΔCt method was used to calculate relative expression of each gene.

## Results

### Histology of TRAMP Mouse Prostate

**Figure 1:**
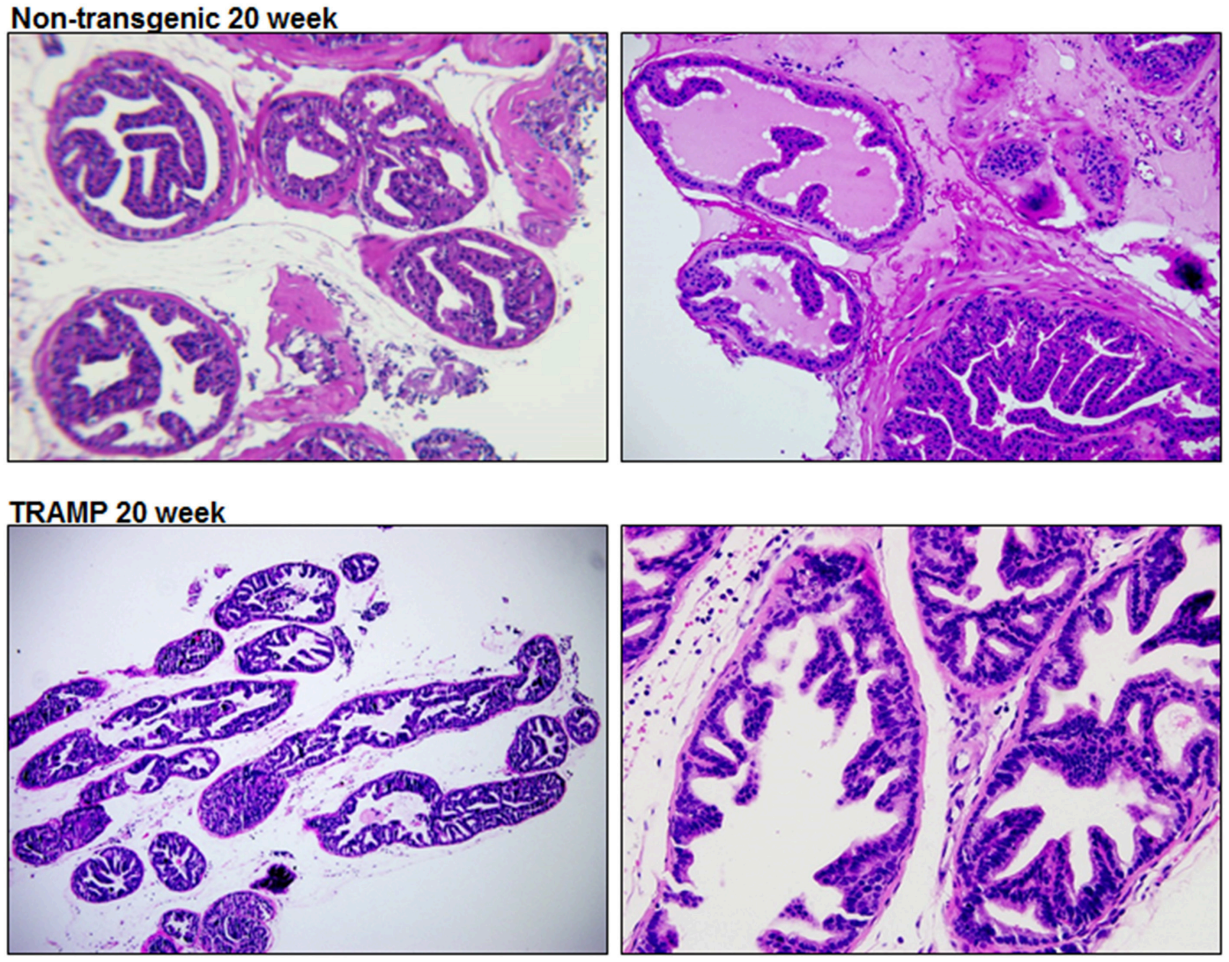
Histology of dorsolateral prostate of TRAMP mice and non-transgenic littermates. Representative staining image of histopathology of 20 week old TRAMP mice and non-transgenic littermate. A typical dorsolateral prostate from a non-transgenic mouse exhibited acini with abundant eosinophilic intraluminal secretions. TRAMP mouse exhibited adenocarcinoma with extensive epithelial stratification, crowded cribriform structures accompanied with marked thickening, remodeling and hypercellularity of the fibromuscular stroma. (Magnification x20 and x40).

TRAMP mice is extensively used to discover the roles played by specific pathways, transcription factors, or metabolic pathways in prostate cancer progression (Morgenbesser et al., 2007; Haram et al., 2008; Zhang et al., 2011; Li et al., 2018). Hundred percent of TRAMP mice develop prostate cancer that progresses to poorly differentiated disease if left untreated. The histologic findings and pathological grading in TRAMP mice has been previously documented (Kaplan-Lefko et al., 2003; Berman-Booty et al., 2012). Typically, 20 week dorsolateral prostate of TRAMP mouse consists of poorly differentiated PIN characterized by profound cribriform structures and numerous apoptotic bodies, invasive glands accompanied by marked thickening, remodeling, and hypercellularity of the fibromuscular stroma. Malignant lesions were detected in 40–50% abundance in the dorsolateral prostates of TRAMP mouse consisting of well-differentiated adenocarcinoma (30–40%), along with moderately-differentiated cancer (5–10%) and, infrequently, poorly-differentiated adenocarcinoma (< 1%). Approximately 40–50% of the prostate was non-cancerous and did not exhibit detectable cancer lesions. Histologic evaluation of the dorsolateral prostate from age-matched non-transgenic littermate demonstrated acini with abundant eosinophilic intraluminal secretions. The acini are lined by a layer of well-organized columnar secretory epithelium possessing round and inconspicuous nuclei. A single layer of thin, flat basal epithelial cells with elongated nuclei typically surrounds the columnar epithelium, and a fibromuscular stroma containing three to four cell layers of stratified smooth muscle surrounding the acinus (Figure 1).

### Microarray Analysis

Pairwise statistical analysis of microarray data of 20 week old TRAMP mice and age-matched non-transgenic littermates revealed differentially expressed genes which were upregulated /downregulated with 1.5 fold cut-off. Out of 35,558 transcripts, 136 transcripts passed the stringent criteria cut-off of 1.5-fold differential expression; more precisely, 104 transcripts exhibited increased and 32 demonstrated decreased expression. Among them, Transglutaminase 4 (TGM4) expression was markedly decreased (log2−1.9) whereas significant increase in the expression (log2 1.12) of ribosomal protein L22 (RPL22) was noted. More interestingly, the expression of majority of RPs transcript showed increased expression in the dataset along with some additional genes. The expression of other differentially expressed genes are shown in Table 1.

**Table 1:** Detailed list of gene sets significantly enriched in microarray gene expression.

**Gene name**	**Non-transgenic mice (log_**2**_)**	**TRAMP mice (log_**2**_)**	**TRAMP vs. Non-transgenic log_**2**_ fold change**	**Differential regulation status**
TGM4	8.067576	6.095323	−1.972253	DOWNREG IN TRAMP
SOSTDC1	6.28833	5.377198	−0.911132	DOWNREG IN TRAMP
MPO	6.261636	5.35168	−0.909956	DOWNREG IN TRAMP
SPRR1A	7.303926	6.450634	−0.853292	DOWNREG IN TRAMP
KLF4	5.972053	5.12466	−0.847393	DOWNREG IN TRAMP
RNU3B1	8.152078	7.359946	−0.792132	DOWNREG IN TRAMP
RNU3B3	8.152078	7.359946	−0.792132	DOWNREG IN TRAMP
RNU3B4	8.152078	7.359946	−0.792132	DOWNREG IN TRAMP
MT-ATP6	11.152495	10.3733	−0.779195	DOWNREG IN TRAMP
MT-ATP8	11.152495	10.3733	−0.779195	DOWNREG IN TRAMP
TMEM170A	7.168522	6.393305	−0.775217	DOWNREG IN TRAMP
LYNX1	6.509217	5.743623	−0.765594	DOWNREG IN TRAMP
IFNL3	7.614195	6.864119	−0.750076	DOWNREG IN TRAMP
PPP1R3G	6.619849	5.871467	−0.748382	DOWNREG IN TRAMP
GPR15	6.228328	5.501892	−0.726436	DOWNREG IN TRAMP
UGT2A3	4.911755	4.199168	−0.712587	DOWNREG IN TRAMP
MED13L	6.879517	6.175366	−0.704151	DOWNREG IN TRAMP
DNAJC10	5.724132	5.050291	−0.673841	DOWNREG IN TRAMP
NPY	7.177505	6.52748	−0.650025	DOWNREG IN TRAMP
XIST	4.846425	4.21413	−0.632295	DOWNREG IN TRAMP
LST1	6.934321	6.30468	−0.629641	DOWNREG IN TRAMP
WNT9A	6.878282	6.249191	−0.629091	DOWNREG IN TRAMP
LRRN3	5.589029	4.959979	−0.62905	DOWNREG IN TRAMP
GPR45	5.821262	5.199841	−0.621421	DOWNREG IN TRAMP
IFIT1	5.804034	5.18294	−0.621094	DOWNREG IN TRAMP
YWHAQ	5.732451	5.121101	−0.61135	DOWNREG IN TRAMP
KIR3DL1	5.551242	4.945612	−0.60563	DOWNREG IN TRAMP
EPPIN	6.819009	6.216168	−0.602841	DOWNREG IN TRAMP
CRLF1	6.024841	5.423862	−0.600979	DOWNREG IN TRAMP
MAL2	5.271787	4.675268	−0.596519	DOWNREG IN TRAMP
MAP3K15	5.447115	4.854145	−0.59297	DOWNREG IN TRAMP
H1FX	6.468248	5.876241	−0.592007	DOWNREG IN TRAMP
TOX4	3.892922	4.479661	0.586739	UPREG IN TRAMP
RPL38	6.60822475	7.19496625	0.5867415	UPREG IN TRAMP
LSM12	6.117599	6.705587	0.587988	UPREG IN TRAMP
RPL5	6.2154714	6.8039176	0.5884462	UPREG IN TRAMP
HAX1	6.001432	6.590249	0.588817	UPREG IN TRAMP
ALPI	5.728489	6.318577	0.590088	UPREG IN TRAMP
TCTE3	6.1099925	6.7001345	0.590142	UPREG IN TRAMP
RPL7	6.5213525	7.11150425	0.59015175	UPREG IN TRAMP
MRTO4	6.06266	6.6569265	0.5942665	UPREG IN TRAMP
HDAC1	7.1222465	7.7184935	0.596247	UPREG IN TRAMP
UBE2H	5.400706	5.9973295	0.5966235	UPREG IN TRAMP
KRT26	4.576418	5.173867	0.597449	UPREG IN TRAMP
TNFSF10	4.968375	5.566245	0.59787	UPREG IN TRAMP
DPPA2	5.2919825	5.8913585	0.599376	UPREG IN TRAMP
PNLDC1	5.567492	6.168421	0.600929	UPREG IN TRAMP
MRPS11	5.715828	6.317477	0.601649	UPREG IN TRAMP
PFDN6	5.868045	6.472116	0.604071	UPREG IN TRAMP
STOML2	5.471422	6.076051	0.604629	UPREG IN TRAMP
VDAC1	4.739057	5.344863	0.605806	UPREG IN TRAMP
RPS8	8.219871929	8.829568	0.609696071	UPREG IN TRAMP
TDGF1	6.079579	6.690292	0.610713	UPREG IN TRAMP
UBE2E2	5.919762	6.531531	0.611769	UPREG IN TRAMP
GKAP1	4.673868	5.286005	0.612137	UPREG IN TRAMP
RPL10A	6.271725333	6.884766333	0.613041	UPREG IN TRAMP
RPS15	6.904076667	7.518351667	0.614275	UPREG IN TRAMP
UMOD	5.965413	6.58144	0.616027	UPREG IN TRAMP
RPL9	7.8632155	8.47986875	0.61665325	UPREG IN TRAMP
RPL30	7.4728119	8.0894858	0.6166739	UPREG IN TRAMP
NPNT	5.948929	6.566422	0.617493	UPREG IN TRAMP
NR1I2	5.126106	5.744132	0.618026	UPREG IN TRAMP
KRTAP16-1	4.635846	5.256192	0.620346	UPREG IN TRAMP
H3F3A	6.632576333	7.259209167	0.626632833	UPREG IN TRAMP
H3F3B	6.632576333	7.259209167	0.626632833	UPREG IN TRAMP
MRPL34	5.782111	6.409365	0.627254	UPREG IN TRAMP
CSE1L	5.137071	5.76598	0.628909	UPREG IN TRAMP
ST6GALNAC1	6.264418	6.896253	0.631835	UPREG IN TRAMP
UBE2Q2	4.944425	5.578387	0.633962	UPREG IN TRAMP
RPS17	6.891415333	7.531757333	0.640342	UPREG IN TRAMP
RPL35	7.432499	8.07315375	0.64065475	UPREG IN TRAMP
GMCL1	4.814764	5.463716	0.648952	UPREG IN TRAMP
NIP7	4.865122	5.516629	0.651507	UPREG IN TRAMP
MSL2	6.330507	6.982406	0.651899	UPREG IN TRAMP
EIF4A1	6.357854333	7.015451	0.657596667	UPREG IN TRAMP
LASP1	6.2246	6.885856	0.661256	UPREG IN TRAMP
LUZP4	5.420406273	6.082919364	0.662513091	UPREG IN TRAMP
EIF1AD	5.398592	6.061553	0.662961	UPREG IN TRAMP
TUBB3	5.118307	5.782439	0.664132	UPREG IN TRAMP
RPL34	6.812256125	7.48376675	0.671510625	UPREG IN TRAMP
RPL17	8.6381411	9.3102862	0.6721451	UPREG IN TRAMP
RPL23	8.6381411	9.3102862	0.6721451	UPREG IN TRAMP
RPL28	7.3877255	8.060629	0.6729035	UPREG IN TRAMP
KIR3DL2	4.490148	5.163333	0.673185	UPREG IN TRAMP
LMO2	6.194375	6.867663	0.673288	UPREG IN TRAMP
NA	8.854485667	9.528207	0.673721333	UPREG IN TRAMP
AOC3	4.851769	5.52668	0.674911	UPREG IN TRAMP
H2AFZ	5.7490965	6.4253375	0.676241	UPREG IN TRAMP
RPL37	6.697474	7.3776735	0.6801995	UPREG IN TRAMP
TMSB10	6.4661765	7.1498185	0.683642	UPREG IN TRAMP
AMY2A	5.5242838	6.2095316	0.6852478	UPREG IN TRAMP
AMY2B	5.5242838	6.2095316	0.6852478	UPREG IN TRAMP
PPIA	7.218713167	7.904503667	0.6857905	UPREG IN TRAMP
PPIH	7.96199975	8.650384	0.68838425	UPREG IN TRAMP
BEGAIN	5.547309	6.236008	0.688699	UPREG IN TRAMP
NT5C3A	5.18496	5.88516	0.7002	UPREG IN TRAMP
RPS28	7.77441325	8.480784583	0.706371333	UPREG IN TRAMP
RPL27	7.019244167	7.728438	0.709193833	UPREG IN TRAMP
IL24	5.822699	6.533534	0.710835	UPREG IN TRAMP
COPS9	5.550281	6.267586	0.717305	UPREG IN TRAMP
NEK2	5.869081	6.58744	0.718359	UPREG IN TRAMP
SOCS2	5.472304	6.193221	0.720917	UPREG IN TRAMP
UBL5	5.35799	6.081819	0.723829	UPREG IN TRAMP
UBE2D3	6.4919555	7.218215	0.7262595	UPREG IN TRAMP
VHL	5.126836	5.855295	0.728459	UPREG IN TRAMP
RPL23A	7.075150533	7.805176367	0.730025833	UPREG IN TRAMP
AICDA	4.75671	5.493925	0.737215	UPREG IN TRAMP
LDHA	6.949646	7.687545	0.737899	UPREG IN TRAMP
CNEP1R1	5.093965	5.834359	0.740394	UPREG IN TRAMP
RPL29	9.731314667	10.47313587	0.7418212	UPREG IN TRAMP
SPCS1	6.360776	7.105179	0.744403	UPREG IN TRAMP
RPL41	9.0427918	9.7879746	0.7451828	UPREG IN TRAMP
SP110	7.431632667	8.17828	0.746647333	UPREG IN TRAMP
PMP22	7.431632667	8.17828	0.746647333	UPREG IN TRAMP
ARCN1	5.1359	5.882582	0.746682	UPREG IN TRAMP
UQCRB	5.850610667	6.602469667	0.751859	UPREG IN TRAMP
SLC25A19	6.694142722	7.446046167	0.751903444	UPREG IN TRAMP
UBE2N	4.750683	5.50388	0.753197	UPREG IN TRAMP
SNRPE	5.56195	6.319007	0.757057	UPREG IN TRAMP
ACKR1	5.301382	6.059632	0.75825	UPREG IN TRAMP
RPL21	8.995938667	9.774761333	0.778822667	UPREG IN TRAMP
LEFTY2	4.06356	4.843924	0.780364	UPREG IN TRAMP
RPL31	6.676469667	7.475938333	0.799468667	UPREG IN TRAMP
RPL35A	7.471309667	8.272641333	0.801331667	UPREG IN TRAMP
RPL7A	8.902748222	9.712848259	0.810100037	UPREG IN TRAMP
RPL39	5.712307	6.524483	0.812176	UPREG IN TRAMP
NACA	6.618499	7.4467945	0.8282955	UPREG IN TRAMP
SP140	7.564562	8.408509	0.843947	UPREG IN TRAMP
MRPL23	5.8653735	6.718201	0.8528275	UPREG IN TRAMP
VDAC3	4.719043333	5.638291667	0.919248333	UPREG IN TRAMP
VDAC1P5	4.719043333	5.638291667	0.919248333	UPREG IN TRAMP
ATP5L	6.3129462	7.2759266	0.9629804	UPREG IN TRAMP
DIMT1	4.137816	5.183852	1.046036	UPREG IN TRAMP
GMCL1P1	6.046781	7.0991925	1.0524115	UPREG IN TRAMP
ARFGAP2	6.024623	7.141176	1.116553	UPREG IN TRAMP
RPL22	5.681224	6.802828	1.121604	UPREG IN TRAMP

### Validation of Microarray Data Using qRT-PCR

**Figure 2:**
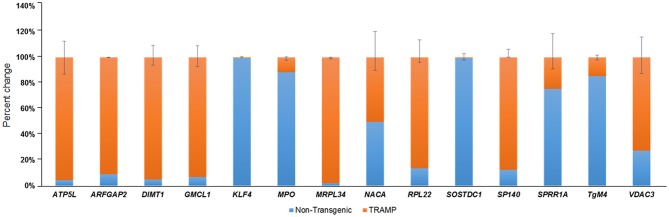
Transcriptional changes of select number of transcripts identified by microarray in non-transgenic and TRAMP mice and their validation by qRT-PCR. Percent change in ATP5L, ARFGAP2, DIMT1, GMCL1, KLF4, MPO, MRPL34, NACA, RPL22, SOSTDC1, SP140, SPRR1A, TgM4, and VDAC3 genes are shown. Values represented in the graph are percentage (%) change of transcript abundance between TRAMP mice compared with non-transgenic littermates. Error bars are standard error of percent changes driven from three biological and technical replicates. The orange color in the graph represents the expression of genes in TRAMP mice compared to blue which represent the non-transgenic mice gene expression. Mean ± standard error. Student's *t*-test for independent groups; *p* < 0.01; (*n* = 3).

In order to confirm findings of the microarray data, we selected a subset of 14 genes which are differentially regulated in TRAMP mice compared with non-transgenic littermates. These genes were evaluated by qRT-PCR. Among the differentially expressed genes, the relative percentage expression of 8 genes, including *ATP5L, ARFGAP2, DIMT1, GMCL1, MRPL34, RPL22, SP140*, and *VDAC3* were significantly upregulated in the dorsolateral prostate of TRAMP mice by 80–100%. In contrast, the expression of 5 genes viz. *KLF4, MPO, SOStdc1, SPRR1A*, and *TGM4* were significantly downregulated in the dorsolateral prostates of TRAMP mice at *p* < 0.01. However, the expression of *NACA* gene remained unaltered in both non-transgenic and TRAMP mice (Figure 2).

### Protein Classification

**Figure 3:**
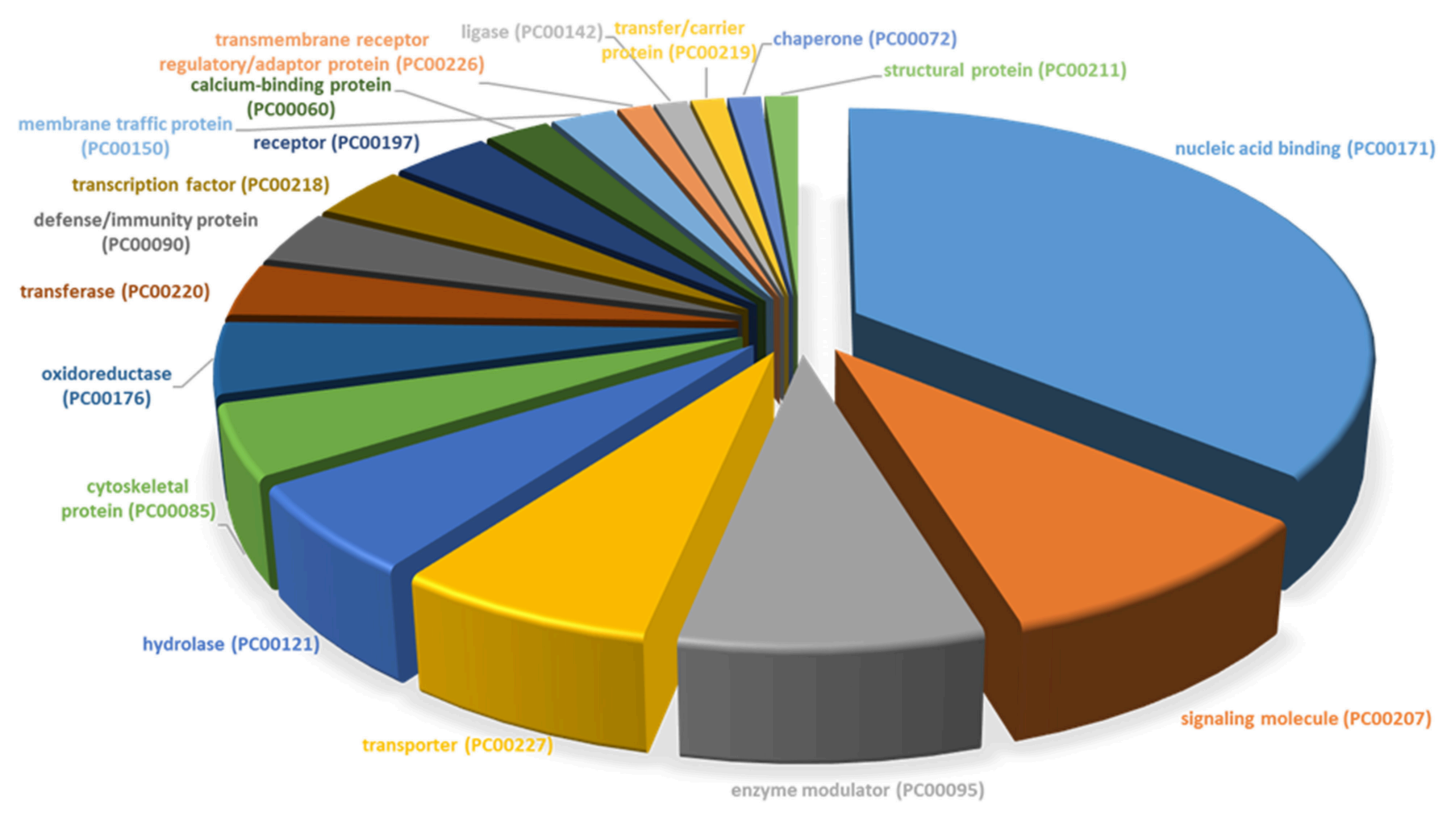
PANTHER protein class categories of total DEGs in TRAMP mice and non-transgenic littermates. Pie chart of the broad biological functions associated with differentially expressed proteins in the prostates of TRAMP mice and non-transgenic littermates. Protein classes were grouped by the protein classification tool in PANTHER and major functions were categorized using human Gene Ontology and PANTHER with detailed category.

All differentially expressed genes in TRAMP mice (*n* = 136), were aligned with human genome database using PANTHER which identified 18 different classes of matched proteins. The extracellular matrix protein identified in TRAMP mice did not match with the human genome database (Table 2). Majority of the matched proteins with human database were nucleic acid binding proteins (~36%), including ribosomal proteins (small and large), and 9% were classified as signaling molecules and 8% as enzyme modulators. Other protein classes identified were transporter proteins (7%), hydrolases (6%), oxidoreductases and cytoskeleton proteins (5%). Less than 5% proteins were characterized as being calcium binding proteins, defense/immunity proteins, transfer/carrier proteins, transmembrane receptor/adaptor proteins, transcription factors, membrane traffic proteins, structural proteins, receptors, ligases, and chaperon proteins (Figure 3).

**Table 2:** Side-by-side comparison of protein class differentially expressed in TRAMP and its homology to human gene ontology.

**S. no**	**Protein class human gene ontology**	**(%) Homology**	**S. no**	**Protein class mouse gene ontology**	**(%) Homology**
1	Nucleic acid binding (PC00171)	36.00%	1	Nucleic acid binding (PC00171)	36.00
2	Signaling molecule (PC00207)	9.30%	2	Signaling molecule (PC00207)	9.30
3	Enzyme modulator (PC00095)	8.10%	3	Enzyme modulator (PC00095)	7.00
4	Transporter (PC00227)	7.00%	4	Transporter (PC00227)	5.80
5	Hydrolase (PC00121)	5.80%	5	Hydrolase (PC00121)	5.80
6	Cytoskeletal protein (PC00085)	4.70%	6	Cytoskeletal protein (PC00085)	4.70
7	Oxidoreductase (PC00176)	4.70%	7	Oxidoreductase (PC00176)	4.70
8	Transferase (PC00220)	3.50%	8	Receptor (PC00197)	4.70
9	Defense/immunity protein (PC00090)	3.50%	9	Transferase (PC00220)	3.50
10	Transcription factor (PC00218)	3.50%	10	Defense/immunity protein (PC00090)	3.50
11	Receptor (PC00197)	3.50%	11	Transcription factor (PC00218)	3.50
12	Calcium-binding protein (PC00060)	2.30%	12	Calcium-binding protein (PC00060)	2.30
13	Membrane traffic protein (PC00150)	2.30%	13	Membrane traffic protein (PC00150)	2.30
14^¶^	Transmembrane receptor regulatory/adaptor protein (PC00226)	1.20%	14	Extracellular matrix protein (PC00102)	1.20
15	Ligase (PC00142)	1.20%	15	Transmembrane receptor regulatory/adaptor protein (PC00226)	1.20
16	Transfer/carrier protein (PC00219)	1.20%	16	Ligase (PC00142)	1.20
17	Chaperone (PC00072)	1.20%	17	Transfer/carrier protein (PC00219)	1.20
18	Structural protein (PC00211)	1.20%	18	Chaperone (PC00072)	1.20
			19	Structural protein (PC00211)	1.20

Column 14

¶ * No match for protein class between mouse and human gene ontology*.

### Canonical Pathway Analysis and Z Score Activation

**Figure 4:**
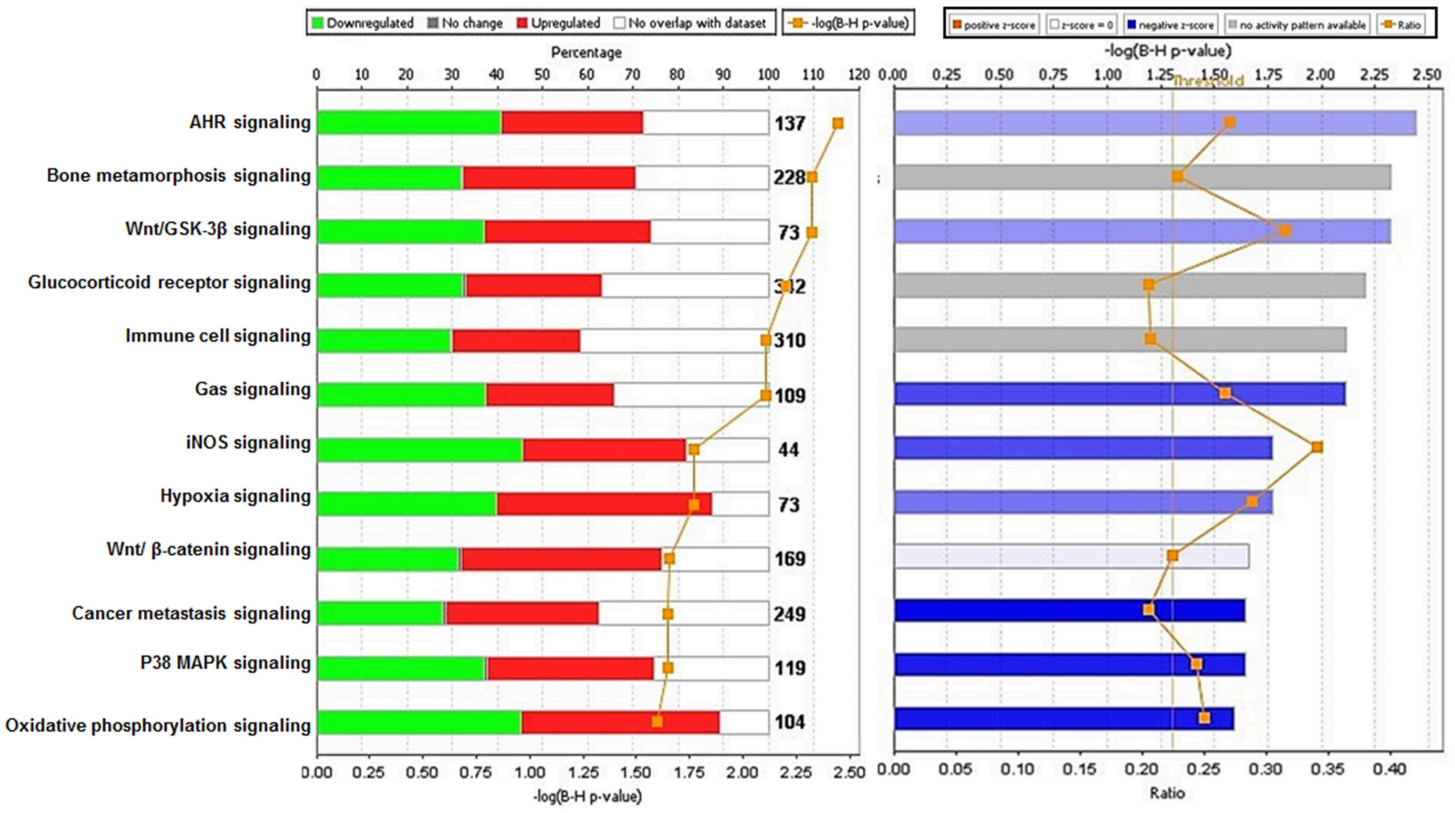
Ingenuity pathway analysis of differentially expressed transcript in TRAMP mice compared with non-transgenic littermates. The stacked bar chart represents the percentage (%) of genes that were upregulated (red), and downregulated (green) and genes not overlapped with TRAMP mouse dataset (white) in each canonical pathway. The numerical value at the top of each bar represents the total number of genes in the canonical pathway. The secondary y-axis (down) shows the –log of *P*-value calculated by the Benjamini-Hochberg (B-H) method; the B-H method was used to adjust the right-tailed Fisher's exact test *P*-value, which indicates the significance of each pathway (left panel graph). The right panel graph calculated z-score indicates a pathway with genes exhibiting decreased mRNA levels (blue bars) or under the state of inhibition (light blue bars). The ratio (orange dots connected by a line) indicates the ratio of genes from the dataset that map to the pathway divided by the total number of genes that map to the same pathway.

Analysis performed using IPA 5.0 (human database) revealed pathways that were significantly overexpressed in TRAMP prostate and demonstrate similarity to human prostate cancer. Twelve pathways common in both human and mouse database were identified and ranked with their respective [-log B-H *p*-value]: (i) Aryl hydrocarbon receptor (AHR) signaling, (ii) Bone metamorphosis signaling, (iii) Wnt/GSK-3β signaling, (iv) Glucocorticoid receptor signaling, (v) Immune cell signaling, (vi) Gas signaling, (vii) iNOS signaling, (viii) Hypoxia signaling, (ix) Wnt/β-catenin signaling, (x) Cancer metastasis signaling (xi) p38MAPK signaling, and (xii) Oxidative phosphorylation (Figure 4-left panel). The activation score (Z) was analyzed with IPA software and with reference to the score it was presumed that all 12 overrepresented pathways were either inhibited (dark blue) or under the state of inhibition (light blue) (Figure 4-right panel). All 12 overrepresented pathways were overlaid with cancer, and the molecules associated with malignancy were shown in pink circle, and those with upregulation/downregulation is shown in red/green (Supplemental Figures 1–12).

### Interactive Network Analysis

**Figure 5:**
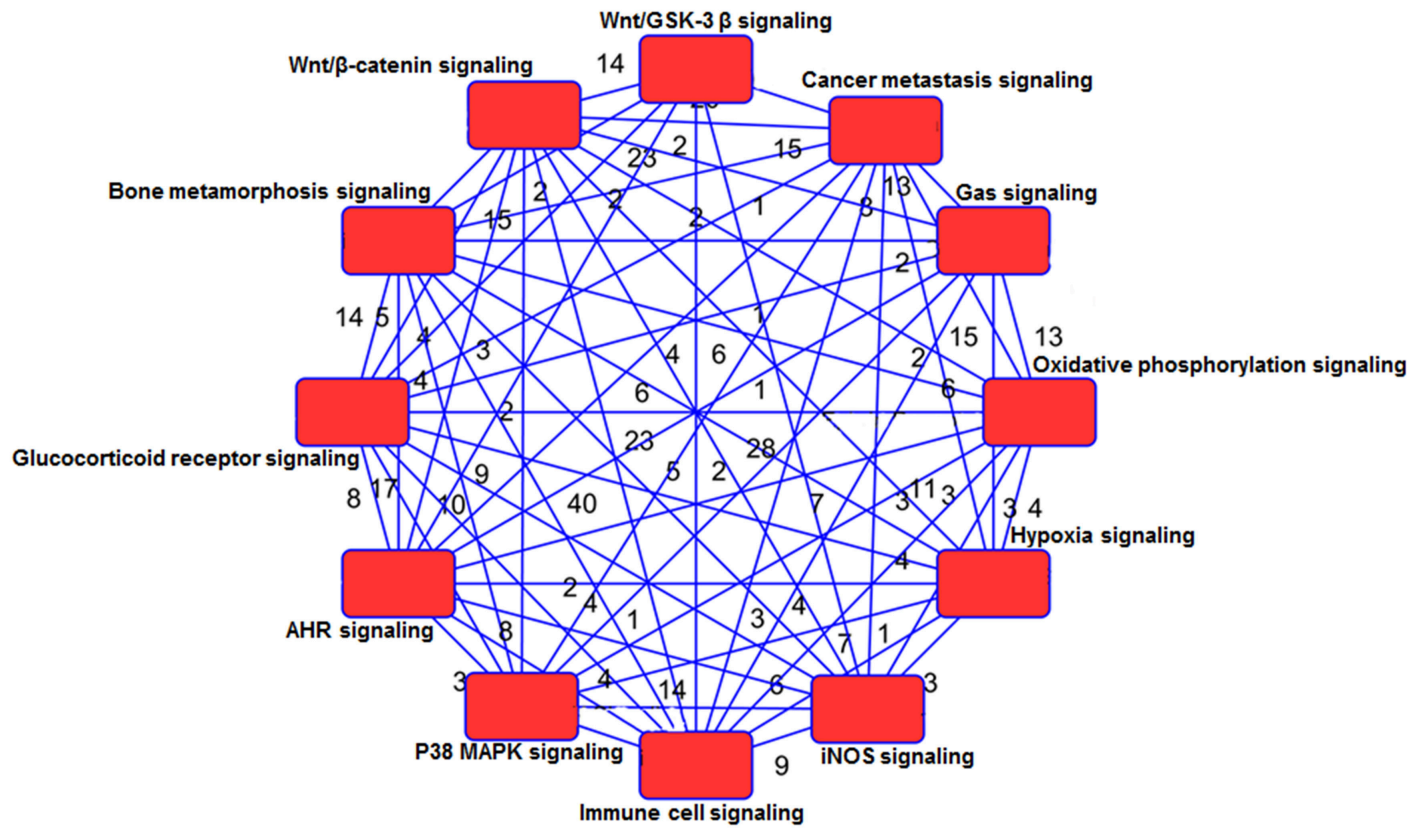
Network analysis of genes and shared pathways in TRAMP mice. The red box depicts signaling pathways and the numerical values represent genes associated with other signaling network.

Using IPA 5.0, we studied the role of differentially expressed genes in TRAMP mice and their interaction in driving prostate carcinogenesis by generating networks of interacting genes. Each network was ranked on the basis of number of differentially expressed genes (Table 3) and mapped to the human network in ingenuity knowledge base and size of the network (Figure 5).

**Table 3:** Genes associated with canonical pathways and network in prostate cancer.

**Molecules in canonical pathways**	**Ratio**	**Genes associated with canonical pathways**
Aryl hydrocarbon receptor signaling	5.19	HSPB3, NFIX, GSTT1, ARNT, GSTZ1, CTSD, HSP90B1, JUN, SP1, NCOA2, ALDH1A3, MAPK3, NEDD8, TGFB2, AHR, GSTA3, SRC, MGST1, CYP1A1, NFIC, TYR, GSTM3, NQO1, APAF1, MAPK8, ALDH8A1, BAX, NCOA3, ALDH9A1, AHRR, ALDH1L2, ALDH1A2, IL1B, ALDH3B1, DCT, ALDH16A1, MCM7
Bone metamorphosis signaling	4.66	HMP15, NFATC3, TAB2, CSNK1A1, WNT16, IL18R1, IKBKG, ITGA3, MAPK3, WNT7B, DKK2, IRS2, GSK3B, NFKBIB, BIRC3, WNT5B, TNFRSF11B, SPP1, WNT9A, TCF3, IL33, IL1RN, DKK4, IL1RAPL1, TCF4, IL1RL1, BMP3, LRP6, WNT8B, WNT2, JUN, NFAT5, AKT3, ALPL, SRC, IFNG, IL1RAPL2, GRB2, WNT2B, ITGA2, MAPK8, TCF7L1,IL36B,TRAF2,CSF1,IL1B,BMP7,DKK1,TCF7L2,LRP1,WNT11,IL11
Wnt/GSK-3β signaling	4.61	HFNG, TCF4, IFNE, WNT9A, WNT2B, IFNB1, CSNK1A1, WNT16, IFNA4, TCF7L1, TCF3, IFNA14, WNT8B, NCOA3, WNT2, NCOA2, WNT7B, NCOA1, GSK3B, IFNK, WNT5B,WNT11,TCF7L2
Glucocorticoid receptor signaling	4.35	HSPA14, NFATC3, HSPA5, HSPA4, CXCL3, IKBKG, KRT13, BAG1, MAPK3, SUMO1, KRT79, MRAS, IRS2, NFKBIB, TAB1, KRT31, KRT78, MED14, NCOA3, TAF4B, TAF1, KRT27, TAF5, TAF4, IL1RN, KRT19, NCOA1, KRT24, KRT18, KRT15, KRT34, ARID1A, IL5, KRT76, JAK2, MAPK11, HSPA1L, HSP90B1, NFAT5, JUN, AR, NCOA2, TGFB2, AKT3, KRT74, KRT20, NOS2, STAT1, MAP2K1, GTF2H3, KRT14, IFNG, IL3, VCAM1, KRT25, GRB2, TAF5L, MAPK8, KRT5 KRT35, TRAF2, GTF2H4, GTF2E1, IL1B, KRT6A, NPPA, PTGS2, JAK3, ELK1, TAF15
Immunity cell signaling	4.15	HOCS1, SOCS3, TRAF3, NFATC3, TLR8, CSNK1A1, IL17RC, WNT16, IL18R1, VEGFA, IKBKG, MAPK3, WNT7B, MRAS, DKK2, ATF4, IRS2, GSK3B, LTBR, NFKBIB, WNT5B, TNFRSF11B, MIF, WNT9A, TCF3, ATF2, IL33, IL1RN, DKK4, IL1RAPL1, TCF4, IL1RL1, LRP6, CEBPZ, JAK2, IL17RA, WNT8B, WNT2, IRAK1, NLK, JUN, NFAT5, AKT3, NOS2, MAP2K1, SRC, VCAM1, IL1RAPL2, GRB2, CREB3L3, IL15, WNT2B, TCF7L1, IL36B, TLR4, TRAF2, CSF1, IL1B, DKK1, TCF7L2, LRP1, WNT11, IRAK4, IRAK2
Gas signaling	4.08	HTR5A, HTR4, ADCY4, RACK1, LHCGR, GNG13, GNB4, MAPK3, MRAS, ATF4, ADORA2B, ADCY8, MAP2K1, GNG4, SRC, HRH2, ADD2, ADCY3, CREB3L3, DRD5, GNG3, CRHR1, MC4R, GNG10, ATF2, AVPR2, ADD3, MC1R, ELK1
Hypoxia signaling	3.62	HBE2A, CREB3L3, NQO1, UBE2W, NOS3, UBE2O, UBE2L6, ARNT, ATF2, VEGFA, UBE2L3, IKBKG, HSP90B1, JUN, EDN1, SUMO1, UBE2G1, UBE2K, ATF4, UBE2E3, NFKBIB
iNOS signaling	3.62	HFNG, IFNGR1, JAK2, MAPK11, RAK1, LR4, IKBKG, JUN, JAK3, NOS2, STAT1, NFKBIB, IRAK4, TAB1, IRAK2

### Signaling Pathway and Its Relevance With Human Prostate Cancer

**Figure 6:**
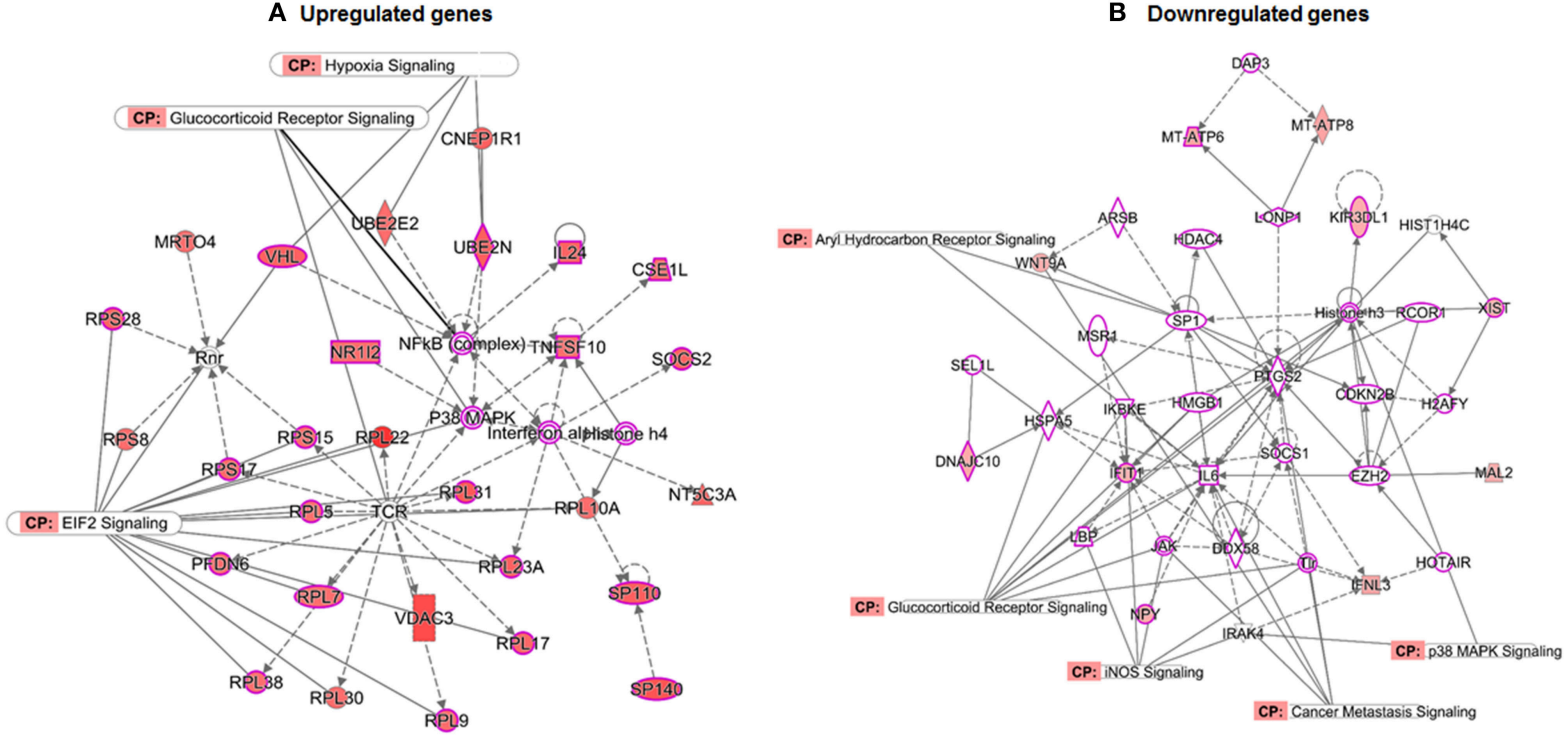
Gene networks and canonical pathways generated by Ingenuity Pathway Analysis. **(A)** Upregulated gene network **(B)** downregulated gene network including proteins and enzymes, G-protein coupled receptors and different regulators and receptors compared between TRAMP mice and non-transgenic littermates. Solid and dotted lines imply direct and indirect relationships between proteins. The intensity of the node color (red) indicated the degree of upregulation and pink color is designated as cancer associated molecules/pathways. The node shapes denote enzymes, phosphatases, kinases, peptidases, G-protein coupled receptor, transmembrane receptor, cytokines, growth factor, ion channel, transporter, translation factor, nuclear receptor, transcription factor.

To examine the interaction of differentially regulated transcripts with overrepresented pathways, we performed network analysis using IPA 5.0. The functional relationship of molecular networks with gene products was based on known connections in peer-reviewed literature. Analysis identified several molecules involved in binding, activation, inhibition and biological processes during prostate cancer progression. As shown in Figure 6A, the ubiquitin genes viz. Ubiquitin-conjugating enzyme E2 E2 (UBE2E2), and UBE2N were upregulated in prostate cancer and linked to hypoxia signaling (Figure 6A). Furthermore, gene expression for ribosomal proteins (RPs) particularly the small subunits viz. RPS7, RPS8, RPS10, RPS13, RPS15A, RPS17, RPS20, RPS28, and RPS29 and large subunits viz. RPL5, RPL7, RPL9, RPL10A, RPL17, RPL19, RPL22, RPL23A, RPL30, RPL31, and RPL38 were upregulated in prostate cancer (Figure 6A). These ribosomal proteins (small and large) are directly associated with Eukaryotic Initiation Factor 2 (eIF2) signaling and small subunit of ribosomal proteins including RPS8, RPS28, RPS17, and RPS15 showed indirect interaction with Ribonucleotide reductase (Rnr). The Rnr signaling pathway regulates dNTP production and influences DNA replication fidelity in majority of eukaryotic cells. Moreover, the von Hippel-Lindau (VHL), classified as a tumor suppressor gene, is directly linked with Rnr and hypoxia signaling. While the Glucocorticoid receptor signaling pathway linked directly with NF-κB complex, p38MAPK and T-cell receptors and indirectly associated with interferons (IL24), Tumor Necrosis Factor (Ligand) superfamily, Member 10 (TNFSF10), TNFSF10, and RPL10A directly linking with histone h4 complex during prostate cancer development (Figure 6A).

Analysis of downregulated genes revealed that glucocorticoid receptor signaling is associated with 8 molecules which include Heat Shock Protein Family A (Hsp70) Member 5 (HSPA5), Interleukin 6 (IL6), Janus kinase (JAK), Toll-like receptors (TLRs), inhibitor of NF-κB (IKB) and histone h3 (Figure 6B). Cancer metastasis signaling was linked with JAK kinase, Wnt family member 9A (WNT9A), IL6, and prostaglandin-endoperoxide synthase 2 (PTGS2); whereas interleukin 1 receptor associated kinase 4 (IRAK4), and histone h3 were associated with p38MAKP signaling pathway. Transcription factor Sp1 and interleukin 6 are directly associated with AHR signaling pathway and indirectly linked with suppressor of cytokine signaling 1 (SOCS1). Five molecules which were downregulated in prostate cancer viz. interleukin 1 receptor associated kinase 4 (IRAK4), JAK kinase, lipopolysaccharide binding protein (LBP), Toll-like receptor (TLR), and Interferon-induced protein with tetratricopeptide repeats 1 (IFIT1) were associated with iNOS signaling (Figure 6B).

### Cross-Reference Differentially Expressed Genes in TRAMP and Its Validation in Human Dataset

Using three reported dataset from human prostate cancer vs. human prostate benign tissue microarray (Varambally et al., 2005; Arredouani et al., 2009; Meller et al., 2016), we performed an analysis overlaying TRAMP mice with human dataset to construct a table with top 10 upregulated/downregulated genes. The top 10 genes upregulated in TRAMP mice are TNFSF, RPL22, EIF4, SLC2A, LMO2/3, KRT, RPS21, RPS19, RPL21, and NEK of which all 10 were upregulated in human prostate cancer dataset. Similarly, top 10 genes downregulated in TRAMP mice cross-referenced with human are *PPP1R3, GPR15, GPR45, TME, TGM4, KLF4, LRRN3, WNT, SPRR1A*, and *TMEM* of which 9 out of 10 were downregulated in human prostate cancer dataset (Table 4). It is highly anticipated that these genes may be identified as therapeutic targets for further study using TRAMP model and its validation in human tissue.

**Table 4:** Genes identified by TRAMP and human overlay.

**S. no**.	**Genes name^€^**	**Mouse**	**Human**	**GEO no**.	**Array platform**	**References**
1.	TNFSF	UPREG	UPREG	GSE3325	GPL570	Varambally et al., 2005
2.	RPL22	UPREG	UPREG	GSE55945	GPL570	Arredouani et al., 2009
3.	EIF4	UPREG	UPREG	GSE55945	GPL570	Arredouani et al., 2009
4.	SLC2A	UPREG	UPREG	GSE69223	GPL570	Meller et al., 2016
5.	LMO2/3	UPREG	UPREG	GSE69223	GPL570	Meller et al., 2016
6.	KRT	UPREG	UPREG	GSE3325	GPL570	Varambally et al., 2005
7.	RPS21	UPREG	UPREG	GSE55945	GPL570	Arredouani et al., 2009
8.	RPS19	UPREG	UPREG	GSE55945	GPL570	Arredouani et al., 2009
9.	RPL21	UPREG	UPREG	GSE55945	GPL570	Arredouani et al., 2009
10.	NEK	UPREG	UPREG	GSE55945	GPL570	Arredouani et al., 2009
11.	PPP1R3	DOWNREG	UPREG	GSE3325	GPL570	Varambally et al., 2005
12.	GPR15	DOWNREG	DOWNREG	GSE3325	GPL570	Varambally et al., 2005
13.	GPR45	DOWNREG	DOWNREG	GSE3325	GPL570	Varambally et al., 2005
14.	TME	DOWNREG	DOWNREG	GSE55945	GPL570	Arredouani et al., 2009
15.	TGM4	DOWNREG	DOWNREG	GSE3325	GPL570	Varambally et al., 2005
16.	KLF4	DOWNREG	DOWNREG	GSE3325	GPL570	Varambally et al., 2005
17.	LRRN3	DOWNREG	DOWNREG	GSE69223	GPL570	Meller et al., 2016
18.	WNT	DOWNREG	DOWNREG	GSE69223	GPL570	Meller et al., 2016
19.	SPRR1A	DOWNREG	DOWNREG	GSE3325	GPL570	Varambally et al., 2005
20.	TMEM	DOWNREG	DOWNREG	GSE69223	GPL570	Meller et al., 2016

€ *A list of 20 genes were identified by overlaying three human prostate cancer vs. benign prostate microarray studies with our TRAMP microarray. Ten of 10 genes were confirmed as upregulated whereas nine of 10 genes were downregulated in TRAMP tumors*.

## Discussion

Although research on prostate cancer has made significant progress in the past decade, the pathogenesis of prostate cancer has yet to be fully elucidated due to its complex biological characteristics and variable heterogeneity. As a result, the early detection and treatment of prostate cancer remains a major challenge. Therefore, understanding of molecular mechanisms and its signaling pathways in prostate cancer based on global gene expression through transcriptomics microarray technology has been widely used to trace disease progression and may aid the identification of key target genes or signaling pathways for early diagnosis, treatment, and prognosis.

We used a similar experimental model and microarray platform as documented in previous studies (Haram et al., 2008). However, previous studies were performed on 30 week old TRAMP mice exhibiting poorly-differentiated cancer mimicking advance-stage malignancy in humans. In contrast, our study is based on 20 week old TRAMP mice displaying well-differentiated cancer emulating early-stage disease. This difference in age and cancer stage has a significant impact in overall implication on molecular pathways. The signaling pathways identified in 30 week TRAMP mice using IPA were different and related to genes regulating cell cycle and metastasis (Haram et al., 2008), whereas our study identified overrepresented signaling pathways including Aryl hydrocarbon receptor (AHR), Bone metamorphosis, Wnt/β-catenin/GSK-3β, Glucocorticoid receptor, Immune cell, Gas, iNOS, Hypoxia, p38MAPK, and Oxidative phosphorylation statistically significant at –log (B-H *p*-value) ranging from 5.19 to 3.37 (Figure 4-right panel), calculated by the Benjamini-Hochberg (B-H) method, directly or indirectly linked with prostate cancer in humans. This marks a significant difference in the dataset and offered the current research a uniqueness to further strengthen the bridge in identifying potential target molecules during prostate cancer development.

Our microarray data revealed 136 deregulated transcripts in the dorsolateral prostate of TRAMP mice, of which 104 transcripts were upregulated, and 32 were downregulated, some of which corresponded to altered genes, which were previously documented in human prostate cancer. Among these genes, a majority were categorized as nucleic acid-binding protein, signaling molecules, transporters, and enzyme modulators. Through the proof-of-principle qRT-PCR studies, we confirmed 6 of the 14 altered genes matched with human prostate cancer. These include voltage dependent anion channel 3 (*VDAC3*) (Asmarinah et al., 2014); Wnt antagonist, *SOSTDC1* (Tesfay et al., 2015); transglutaminase 4 (*TgM4*) (Dubbink et al., 1998); *MRPL34*, a component of the mitochondrial ribosome large subunit (Valdagni et al., 2009); myeloperoxidase (*MPO*) (Roumeguere et al., 2012); and Krüppel-like factor 4 (*KLF4*), a transcription factor with divergent functions (Siu et al., 2012). Of these, *VDAC3* and *MRPL34* were validated as significantly upregulated; whereas *SOSTDC1, MPO, KLF4*, and *TgM4* genes were confirmed as downregulated in the prostate of TRAMP mice by qRT-PCR (Figure 2).

In order to understand how genes involved in these essential biological processes interact to drive the molecular signaling through network, we performed IPA 5.0 analysis. The network based analysis identified various protein classes related to human prostate cancer overlaid with the mouse genome (Table 2). We found significant overlapped genes in canonical pathways associated in context to human prostate cancer (Table 3; Figure 4). The top ranked overrepresented pathway [-log(*p*-value 5.19)] includes the Aryl hydrocarbon receptor (AHR), a ligand-activated transcription factor that interacts with multiple signaling pathways during prostate development. AHR suppresses the viability and migration of human prostate cancer LNCaP cells (Yu et al., 2017), and plays pivotal role in cancer by modulating the downstream expression of Mitogen-Activated Protein Kinase Kinase Kinase 12 (MAP3K12) (Yu et al., 2018). AHR plays an important role in cellular homeostasis and disease development, and the Aryl hydrocarbon receptor nuclear translocator (AHRN) forms a complex with ligand bound AHR during cancer and activates Bax, a central cell death regulator, MAPK, Aldehyde dehydrogenases (ALDHs), Glutathione transferase (GST) and others (Supplemental Figure 1). Strong nuclear AHR expression is observed in the invasive phenotype, and an elevated nuclear AHR expression is associated with poor prognosis in human prostate cancer. On the other hand, there are conflicting results that AHR deficiency results in increased susceptibility to prostate tumors. The molecular mechanism of AHR in prostate cancer needs further investigation.

In cancer, cell proliferation is well-described as fluctuation of hypoxia and nutrient deprivation. In this regard, hypoxia (low oxygen conditions) is considered an early event during prostate carcinogenesis and induces genetic, metabolic, and proteome-related changes. Several signaling pathways are reported to be activated in cancer cells under hypoxic conditions including HIF, PI3K/Akt/mTOR, NOX, Wnt/β-catenin, and Hedgehog (Masoud and Li, 2015). These hypoxia-regulated genes are mediated by hypoxia-inducible factor 1 (HIF-1), the activation of HIF1 then activates the downstream target genes such as JUN, SUMO1, and ubiquitin conjugate enzymes (Supplemental Figure 8). The hypoxia-inducible factor 1-α can be upregulated at the protein level via mTOR or at the mRNA level via STAT-3 and NF-κB signaling (Kwon et al., 2017). Deregulation in Wnt/β-catenin signaling pathway have been shown to interact with several nuclear factors to specific transcriptional target which includes; Jun proto-oncogene (c-Jun), LDL receptor related proteins (1 and 6), SRC proto-oncogene, and TGF beta activated kinase 1 (Supplemental Figure 9). We and others have previously demonstrated the role of these signaling pathways in prostate cancer (Shukla et al., 2004, 2005; Horinaga et al., 2005).

Oxidative phosphorylation is an efficient form of mitochondrial respiration by which cells meet their energy needs and known to regulate genes associated with cell death and metabolism. In our study, cytochrome c oxidase (COX10, COX15, and COX17), NADH-dehydrogenase subunit 4 (MT-ND4), cytochrome c oxidase subunit I and subunit II (MT-CO1, and MT-CO2), and cytochrome c oxidase III (MT-CO3) were downregulated (Supplemental Figure 12). Other genes associated in regulating the oxidative phosphorylation pathways include MT-CO2, MT-CO3, mitochondrial encoded cytochrome B (MT-CYB), Mitochondrially Encoded ATP Synthase Membrane Subunit 6 (MT-ATP6), Mitochondrial Encoded NADH: Ubiquinone Oxidoreductase Core Subunit 4 (MT-ND4), cytochrome c oxidase (COX)10, COX6A2, COX17, COX8C, COX7A1, and COX15 and others (Table 3). Considerable evidence has been published in relation to mitochondrial dysfunction and pathogenesis of prostate cancer (Choudhury et al., 2014). Indeed, mutation in mitochondrial DNA play an important role in the etiology of prostate cancer (Petros et al., 2005). Altered expression of MT-CO2/COX2, and MT-ATP6 transcripts are significantly decreased in prostate tumor specimens [Gleason grade 5 (3 ± 2) to 8 (4 ± 4)] with a median of 7 (Abril et al., 2008), and the downregulation pattern of MT-ATP6 genes further supports our TRAMP expression dataset.

The ubiquitin proteasome pathway, conserved from yeast to mammals, is essential for the targeted degradation of short lived proteins in eukaryotic cells (Mogk et al., 2007; Asaoka and Ikeda, 2015). Ubiquitin plays multiple important roles in cellular processes relevant to cancer. Ubiquitination can result in several cellular processes including protein degradation and control protein–protein interactions (Mansour, 2018; Rape, 2018). Ubiquitin-proteasome is involved in prostate cancer in various ways by modulating prostate cancer-related genes/proteins such as androgen receptor (Li et al., 2014), heat shock protein (HSP) 90 (Chen et al., 2016), cyclin-dependent kinase inhibitor p27, cyclin D1 (Zheng et al., 2016), and PTEN (Yang et al., 2017). Some ubiquitin-like modifier proteins are also associated with prostate cancer (Zuo and Cheng, 2009). In fact, the ubiquitin ligase UBE4A inhibits prostate cancer progression by regulating ILEI1 expression, a protein required for metastatic progression in prostate cancer cells (Sun et al., 2017). Ubiquitin associated genes (UBE2A, UBE2W, UBE2O, UBE2L6, UBE2L3, UBE2G1, UBE2K, UBE2E3) were overexpressed and might be signature molecules of this pathway along with JUN, SUMO1, NF-κB and others (Table 3). The expression of ubiquitin UBE2N, UBE2D3, and UBEQ2 were upregulated in TRAMP mice dataset (Table 1).

Innate immunity and inflammation plays critical role in cancer development and progression (Crusz and Balkwill, 2015). In the present study, interleukins (IL18R1, IL33, IL1RN, IL1R1, IL1RAPl2, and IL3636B) and interleukin 1 receptor antagonist (IL1RN, IL33, IL1RL1, IL17RA, IL17RC), LRP1, LRP6 (Mani et al., 2017), suppressor of cytokine signaling-1 (SOCS-1 and SOCS3), Toll-like receptors 8 (TLR8), and others are underrepresented in TRAMP mice (Table 3; Supplemental Figure 5). Previously, the role of TLR-like genes in prostate cancer was reported (Chen et al., 2005). Sun J. et al. (2005); Sun S. Y. et al. (2005) observed multiple SNPs in strong linkage disequilibrium located in TLRs associate with prostate cancer, and later the role of TLRs-linked with innate immunity signaling was established (Kazma et al., 2012). The role of SOCS genes in association with prostate cancer was demonstrated in prostate cancer cells, suggesting that SOCS acts as a negative growth regulator (Neuwirt et al., 2009).

The WNT genes showed crosstalk with Wnt/GSK-3β signaling pathways where WNT genes marked as potent genes interconnected within pathways in prostate cancer, imprints the effect on cancer stem cell proliferation, migration and differentiation (Morris and Huang, 2016). The genes associated with Wnt/β-catenin includes AKT3, JUN, LRP1, WNT16, WNT2, WNT7B WNT5E, WNT9A, WNT2B, WNT11, and SRC. They showed significant crosstalk with other signaling molecules (Table 3; Supplemental Figure 9). Moreover, the SOX genes represented in this pathway include SOX8, SOX10, SOX14, SOX15, SOX17, and SOX18 (Table 3). The gene expression of SOX in human prostate cancer tissue compared with non-cancerous revealed the fact that SOX7, SOX9, and SOX10 were associated with advance-stage prostate cancer, whereas SOX7 and SOX9 were identified as prognostic biomarker in prostate cancer (Zhong et al., 2012). In p38MAPK signaling showed important associated genes regulating the pathways are MAPK11, STAT1, RPS6, MAPKAPk3, and others (Table 3; Supplemental Figure 11). Additional genes associated in cancer metastasis pathway are WNT7B, WNT9A, WNT2, WNT2B, WNT11, WNT7B, LRP6, SRC, JAK2, JAK3, and others (Table 3; Supplemental Figure 9). The aforementioned associated molecules showed crosstalk with other overrepresented signaling pathways (Figure 5).

Steroid receptors, including the Glucocorticoid receptor (GCR), have been proposed to play an essential role in prostate cancer (Kach et al., 2015). The signature molecules associated with GCR signaling pathway in prostate cancer include heat shock proteins (HSP), Keratins (KRT), SUMO1, AKT3, and others (Table 3; Supplemental Figure 4). Among these, HSP90B showed crosstalk with another signaling pathways; hypoxia and AHR; and SUMO1 showed crosstalk with hypoxia signaling pathway. HSP90 interacts with bHLH-PAS domain of HIF-1α which is a major regulator of HIF-1α activation (Minet et al., 1999). With reference to downregulated genes and its interconnectivity with other signaling pathways, the GCR signaling was linked with Hsp70, IL-6, TLR and PTGS2 (Figure 6B). HSP70 implied significant role in prostate cancer by regulating androgen receptor signaling (Kita et al., 2017). Studies have shown overexpression of IL24 in TRAMP mice, possibly interleukin promotes initial steps of prostate cancer progression by upregulating genes such as matrix metallopeptidase 9, (MMP9) *via* the JAK-STAT signaling pathway (Shukla et al., 2007). Moreover, interleukin has also been implicated with cancer progression having ability to mediate osteoblast differentiation (Bellido et al., 1997) and crosstalk with Wnt and JAK signaling pathways (Supplemental Figure 2). Moreover, the role of AHR linked with SP1 and IL warrants further investigation in context to prostate cancer.

Gas signaling molecules including nitric oxide (NO), carbon monoxide (CO), and hydrogen sulfide (H2S) induce reactive oxygen species, which initiate metabolic changes and protein translation in cancer cells, allowing them to survive and facilitate tumor progression. Studies suggest that continuous high concentrations of nitric oxide (NO), produced by inducible NO synthase (iNOS), promotes prostate tumor initiation and lethal progression (Davila-Gonzalez et al., 2017; Erlandsson et al., 2018). Studies have also shown that nitric oxide synthase gene polymorphisms is associated with aggressive prostate cancer (Lee et al., 2009). In human prostate cancer LNCaP cells, NO upregulates RUNX2 and Bcl2 expression which confers resistance to chemotherapy (Nesbitt et al., 2016). Genes associated with iNOS signaling includes Interleukin-1 receptor-associated kinase 1 (IRAK1, IRAK2, IRAK4), JAK2, JAK3, JUN, MAPK11, NF-κB, NOS2, STAT1, TLR4 (Supplemental Figure 7). Crosstalk of gas signaling molecules with other signaling pathways such as JUN, JAK2, JAK3, and NF-κB, upregulates p38MAPK, hypoxia, immune cell, and cancer metastasis signaling (Figure 5; Supplemental Figure 6). Some other genes associated with gas signaling pathway include G Protein Subunit Gamma (GNG4, GNG3, GNG10, GNG13, Guanine nucleotide-binding proteins G proteins-GNB4, MAPK3, MAP2K, and SRC; Table 3). However, the functional role of these genes and their crosstalk with gas signaling is not fully elucidated. Additional studies on these molecules in context to prostate cancer development and/or progression is warranted.

Next, the signaling network further confers the interaction and crosstalk between genes and its associated pathways (Figure 5). NF-κB showed strong interconnectivity with glucocorticoid receptor signaling pathway (Widén et al., 2003), and the role of NF-κB in context to prostate cancer has been extensively studied by our group (Shukla and Gupta, 2004; Vykhovanets et al., 2011; Shukla et al., 2015). Glucocorticoid receptor also showed connectivity with p38MAPK and T cell receptor (TCR), which warrant further investigation in relation to human prostate cancer. The TCR showed interconnectivity with ELF2 signaling pathway, and ELF2 showed connectivity with RPS5, RPS7, RPS8, RPS10, RPS13, RPS15A, RPS17, RPS20, RPS28, and RPS29 and large; RPL5, RPL7, RPL9, RPL10A, RPL17, RPL19, RPL22, RPL23A, RPL30, RPL31, RPL38, interestingly the ribosomal protein was overwhelmed and upregulated in TRAMP mice dataset (Figure 6A), and tempted us to explore the role of ribosomal protein in prostate cancer. In fact, significant increase of RPL19 mRNA expression in a substantial number of prostate tumors suggest its importance as a biological marker (Bee et al., 2006).

In addition to TRAMP mouse prostate genes, we also screened several genes that have previously been found to be deregulated in human prostate tumors. This validation was performed using three reported dataset (Varambally et al., 2005; Arredouani et al., 2009; Meller et al., 2016) on malignant and normal/benign prostate cancer tissue. A group of 20 genes, which were downregulated/upregulated in TRAMP mice, were compared. Out of ten genes, nine genes showed downregulation in both TRAMP mice and human tissue, whereas all ten out of ten genes were upregulated on both species (Table 4). These results suggest significant gene overlap in molecular pathways of prostate cancer development in TRAMP mice and its recapitulation with human disease.

In conclusion, our comprehensive bioinformatics results, including GO, PPI, and pathway analyses, indicated that the underlying mechanisms of prostate cancer development resulted from dysregulation of 12 key molecular signaling and its connectivity to some major pathways and proteins involved in the progression of human prostate cancer. This study also identified some candidate genes that will be further investigated as biomarkers and potential targets for prevention and/or treatment of prostate cancer.

## Data Availability

The TRAMP microarray dataset was submitted to public repository GEO with accession number GSE119205.

## Author Contributions

SV, SS, MP, and SG conceived and designed the experiments. MP, SS, and, SV performed the experiments. SV and SG performed the data analysis and provided intellectual input. MP, GM, and SG contributed reagents, materials, analysis tools; SV and SG wrote the manuscript.

### Conflict of Interest Statement

The authors declare that the research was conducted in the absence of any commercial or financial relationships that could be construed as a potential conflict of interest.

## Supplementary Material

Supplemental Figure 1 Ingenuity pathway interaction network analysis of Aryl hydrocarbon receptor signaling pathway in prostate cancer. Differentially expressed genes between 20 week old dorsolateral prostate of TRAMP mice exhibiting alterations in elements of Aryl hydrocarbon receptor signaling pathway compared to age-matched non-transgenic littermates.

Supplemental Figure 2 Ingenuity pathway interaction network analysis of role of Bone metamorphosis signaling. Differentially expressed genes between 20 week old dorsolateral prostate of TRAMP mice exhibiting alterations in elements of role of osteoblast and chondrocytes network compared to age-matched non-transgenic littermates.

Supplemental Figure 3 Ingenuity pathway interaction network of Wnt/GSK-3β signaling. Differentially expressed genes between 20 week old dorsolateral prostate of TRAMP mice exhibiting alterations in elements of Wnt/GSK-3β signaling pathway compared to age-matched non-transgenic littermates.

Supplemental Figure 4 Ingenuity pathway interaction network analysis of Glucocorticoid receptor signaling pathway. Differentially expressed genes between 20 week old dorsolateral prostate of TRAMP mice exhibiting alterations in elements of Glucocorticoid receptor signaling pathway compared to age-matched non-transgenic littermates.

Supplemental Figure 5 Ingenuity pathway interaction network analysis of role of Immune cell signaling. Differentially expressed genes between 20 week old dorsolateral prostate of TRAMP mice exhibiting alterations in role of macrophage and other immune cells compared to age-matched non-transgenic littermates.

Supplemental Figure 6 Ingenuity pathway interaction network analysis of Gas signaling pathway. Differentially expressed genes between 20 week old dorsolateral prostate of TRAMP mice exhibiting alterations in elements of Gas signaling pathway compared to age-matched non-transgenic littermates.

Supplemental Figure 7 Ingenuity pathway interaction network analysis of iNOS signaling pathway. Differentially expressed genes between 20 week old dorsolateral prostate of TRAMP mice exhibiting alterations in elements of iNOS signaling pathway compared to age-matched non-transgenic littermates.

Supplemental Figure 8 Ingenuity pathway interaction network analysis of hypoxia signaling pathway. Differentially expressed genes between 20 week old dorsolateral prostate of TRAMP mice exhibiting alterations in elements of hypoxia signaling pathway compared to age-matched non-transgenic littermates.

Supplemental Figure 9 Ingenuity pathway interaction network analysis of Wnt/β-catenin signaling pathway. Differentially expressed genes between 20 week old dorsolateral prostate of TRAMP mice exhibiting alterations in elements of Wnt/β-catenin signaling pathway compared to age-matched non-transgenic littermates.

Supplemental Figure 10 Ingenuity pathway interaction network analysis of Cancer metastasis signaling pathway. Differentially expressed genes between 20 week old dorsolateral prostate of TRAMP mice exhibiting alterations in elements of metastasis signaling pathway compared to age-matched non-transgenic littermates.

Supplemental Figure 11 Ingenuity pathway interaction network analysis of p38MAPKsignaling pathway. Differentially expressed genes between 20 week old dorsolateral prostate of TRAMP mice exhibiting alterations in elements of p38MAPKsignaling pathway compared to age-matched non-transgenic littermates.

Supplementary Figure 12 Ingenuity pathway interaction network analysis of oxidative phosphorylation signaling pathway. Differentially expressed genes between 20 week old dorsolateral prostate of TRAMP mice exhibiting alterations in elements of oxidative phosphorylation signaling pathway compared to age-matched non-transgenic littermates.

Supplemental Table 1 List of set of primers of qRT-PCR with sequence (forward and reverse) used in the experiment to validate microarray data.

## Abbreviations

AHR: Aryl hydrocarbon receptor
AHRN: Aryl hydrocarbon receptor nuclear translocator
ALDH: Aldehyde dehydrogenases
B-H: Benjamin-Hochberg
CO: carbon monoxide
COX: cytochrome c oxidase
DEGs: differentially expressed genes
DIM1: dimethyladenosine transferase 1-like
eIF2: Eukaryotic Initiation Factor 2
FOXO: Forkhead box protein O
GCR: Glucocorticoid receptor
Gsk3: Glycogen Synthase Kinase 3
GNB: Guanine nucleotide-binding proteins
GNG: G Protein Subunit Gamma
GO: Gene ontology
GST: Glutathione transferase
GU: genitourinary
H2S: hydrogen sulfide
H4: histone h4
HIF-1: hypoxia-inducible factor 1
HSP: heat shock protein
HSPA5: Heat Shock Protein member 5
IFIT1: Interferon-induced protein with tetratricopeptide repeats 1
IGF: insulin-like growth factor
IL-1R: interleukin 1 receptor antagonist
IL6: interleukin 6
iNOS: inducible NO synthase
IPA: Ingenuity Pathway Analysis
IRAK4: interleukin 1 receptor associated kinase 4
JAK: Janus kinase
KLF4: kruppel-like factor 4
KRT: Keratins
LBP: lipopolysaccharide binding protein
MAP3K12: Mitogen-Activated Protein Kinase Kinase Kinase 12
MAPK: mitogen activated protein kinase
MPO: myeloperoxidase
MRPL34: mitochondrial ribosomal protein L34
MT-ATP: Mitochondrially Encoded ATP Synthase Membrane
MT-CO: Cytochrome C Oxidase
MT-ND4: NADH-dehydrogenase subunit 4
mTOR: mammalian target of rapamycin
Naca: nascent polypeptide-associated complex alpha polypeptide
NF-κB: Nuclear Factor-KappaB
NO: nitric oxide
PANTHER: Protein Analysis through evolutionary relationships
Pb-Tag: probasin-Tag
PI3K: Phosphoinositide 3-kinase
PIN: prostatic intraepithelial neoplasia
PPI: Protein-protein interaction
PTEN: Phosphatase and tensin homolog
PTGS2: Prostaglandin G/H synthase 2
qRT-PCR: quantitative real-time polymerase chain reaction
Rb: Retinoblastoma
Rnr: U3B small nuclear RNA 1
RPL22: ribosomal protein L22
SOCS: suppressor of cytokine signaling
Sostdc1: sclerostin domain containing 1
Sp1: specificity protein 1
SPRR1b: small proline-rich protein 1B
STAT3: Signal transducer and activator of transcription 3
TgM4: transglutaminase 4
TLR: Toll-like receptors
TNFSF10: Tumor Necrosis Factor Superfamily member 10
TRAMP: transgenic adenocarcinoma of the mouse prostate
UBE: ubiquitin conjugating enzyme
UBE2E2: Ubiquitin-conjugating enzyme E2 E2
VDAC3: voltage-dependent anion channel 3
VHL: von Hippel-Lindau
WNT: Wingless-Type MMTV Integration Site Family
WNT9A: Wnt family member 9A.
