# Supplementary figures and images for: Differentially Expressed Genes and Molecular Pathways in an Autochthonous Mouse Prostate Cancer Model
Source: Front Genet. 2019 Mar 26;10:235. doi: 10.3389/fgene.2019.00235 (PMC6445055; doi:10.3389/fgene.2019.00235)

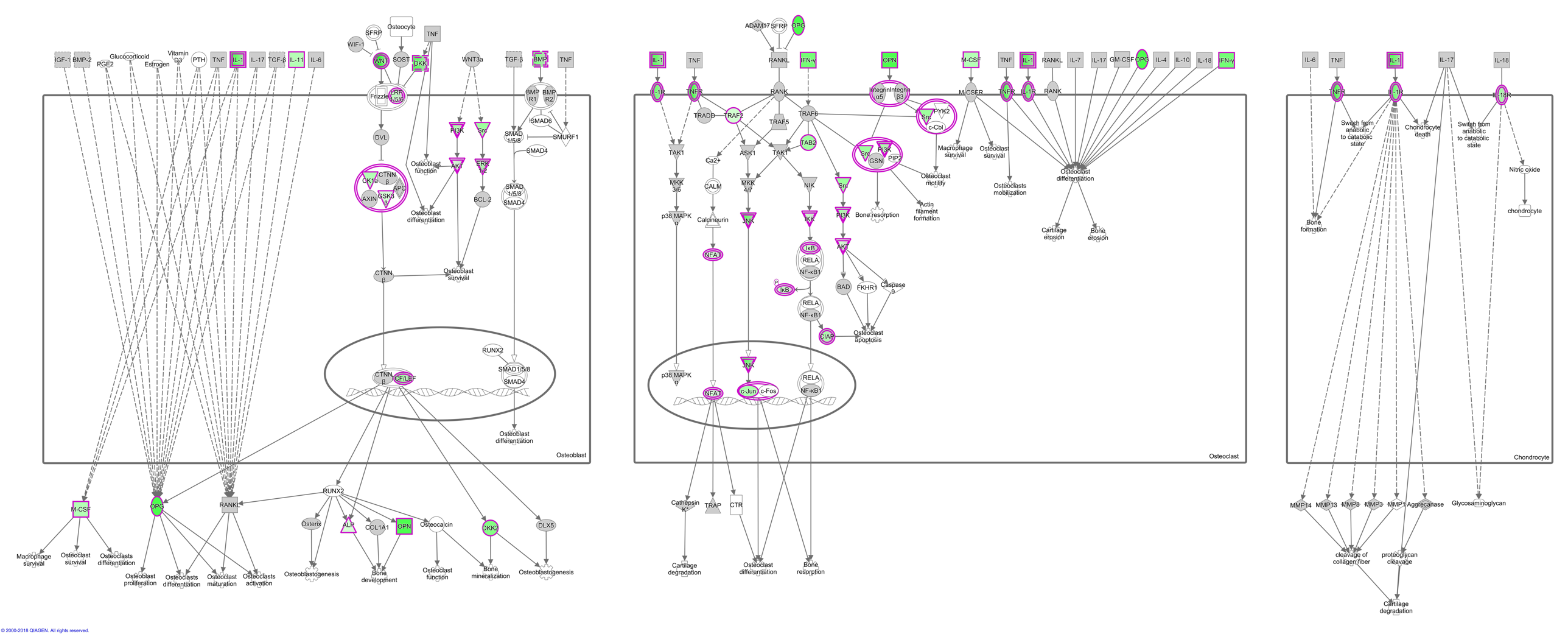

Supplement: Supplemental Figure 2 — Ingenuity pathway interaction network analysis of role of Bone metamorphosis signaling. Differentially expressed genes between 20 week old dorsolateral prostate of TRAMP mice exhibiting alterations in elements of role of osteoblast and chondrocytes network compared to age-matched non-transgenic littermates. [file Image_2.JPEG]

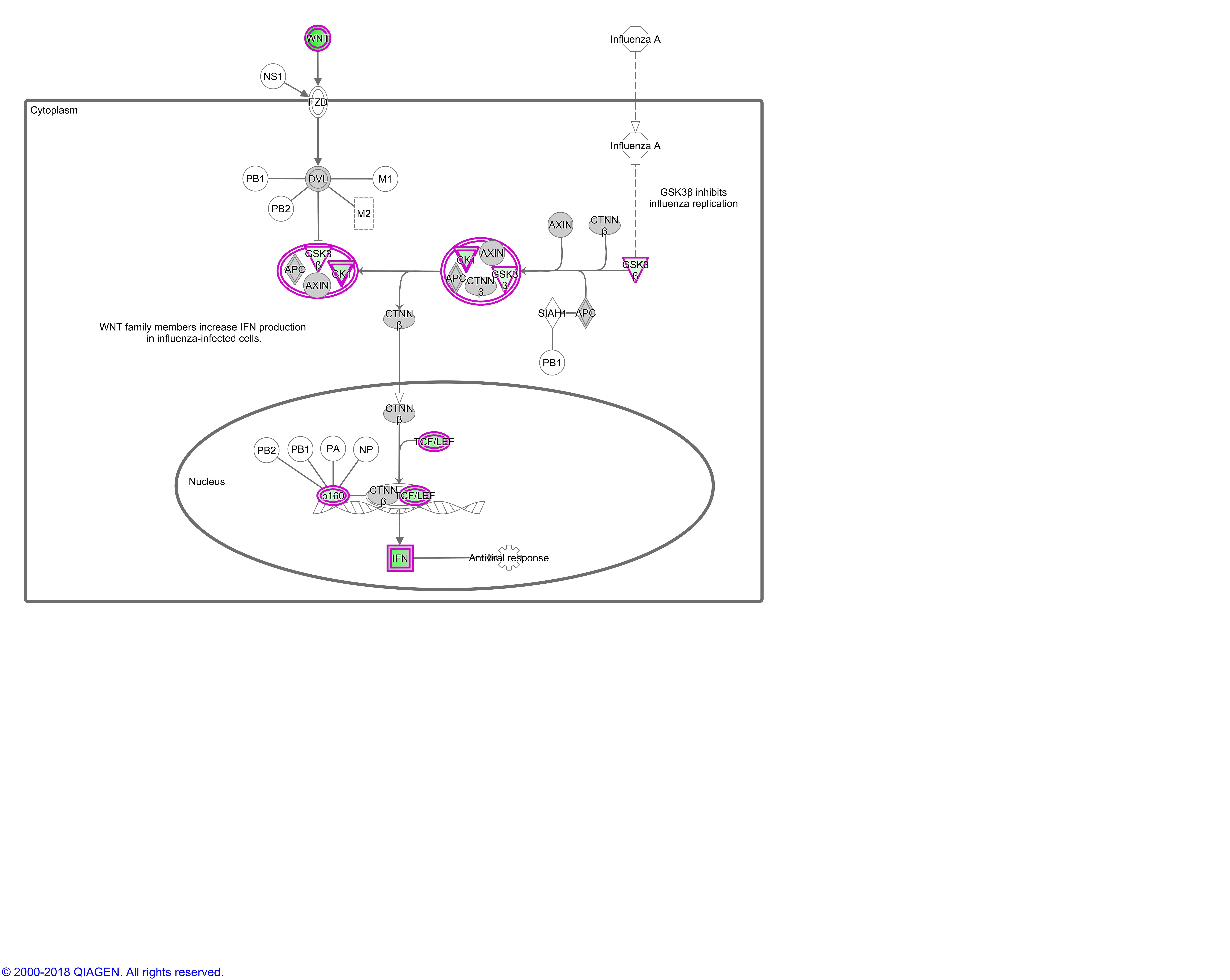

Supplement: Supplemental Figure 3 — Ingenuity pathway interaction network of Wnt/GSK-3β signaling. Differentially expressed genes between 20 week old dorsolateral prostate of TRAMP mice exhibiting alterations in elements of Wnt/GSK-3β signaling pathway compared to age-matched non-transgenic littermates. [file Image_3.JPEG]

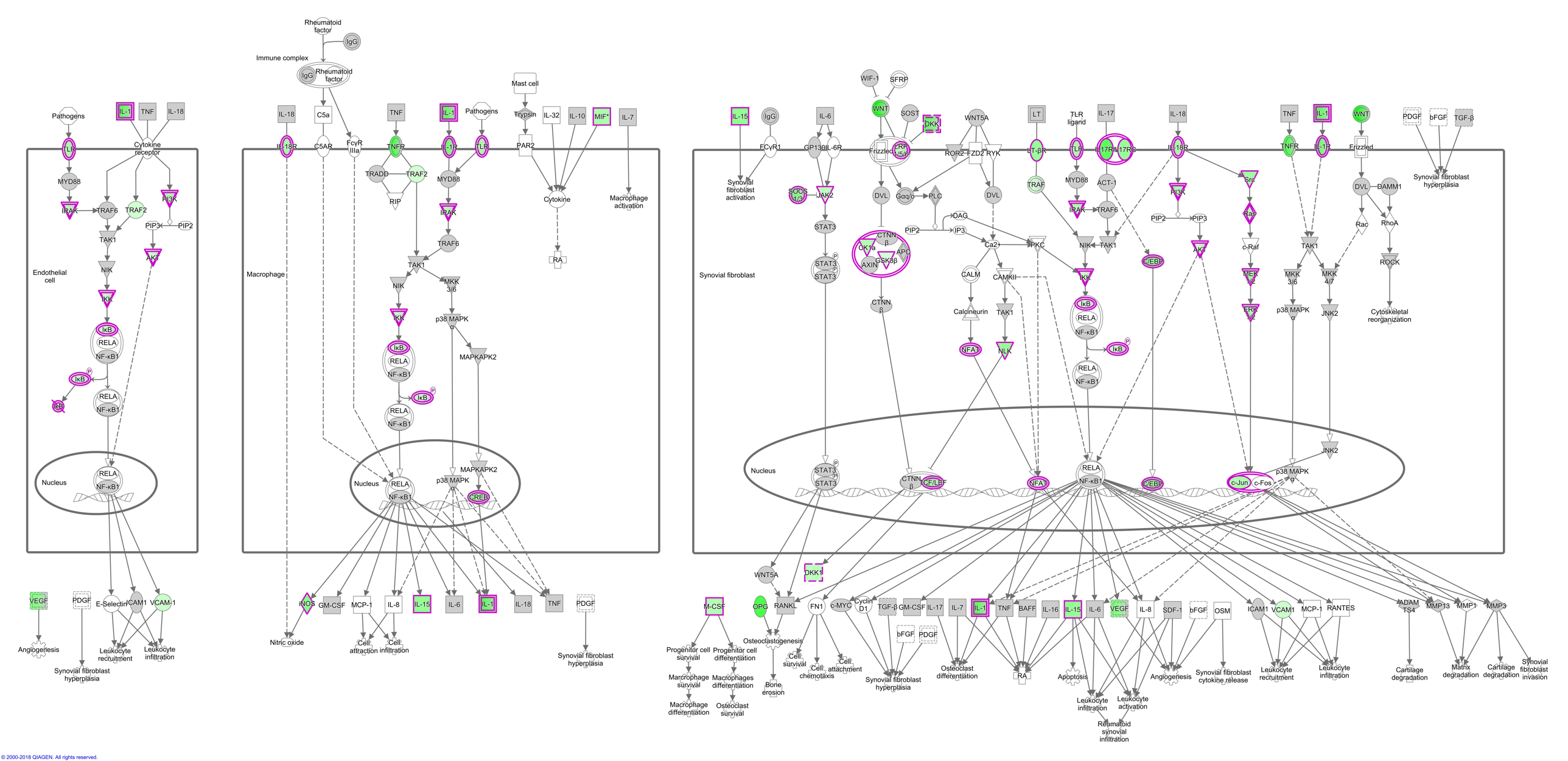

Supplement: Supplemental Figure 5 — Ingenuity pathway interaction network analysis of role of Immune cell signaling. Differentially expressed genes between 20 week old dorsolateral prostate of TRAMP mice exhibiting alterations in role of macrophage and other immune cells compared to age-matched non-transgenic littermates. [file Image_5.JPEG]

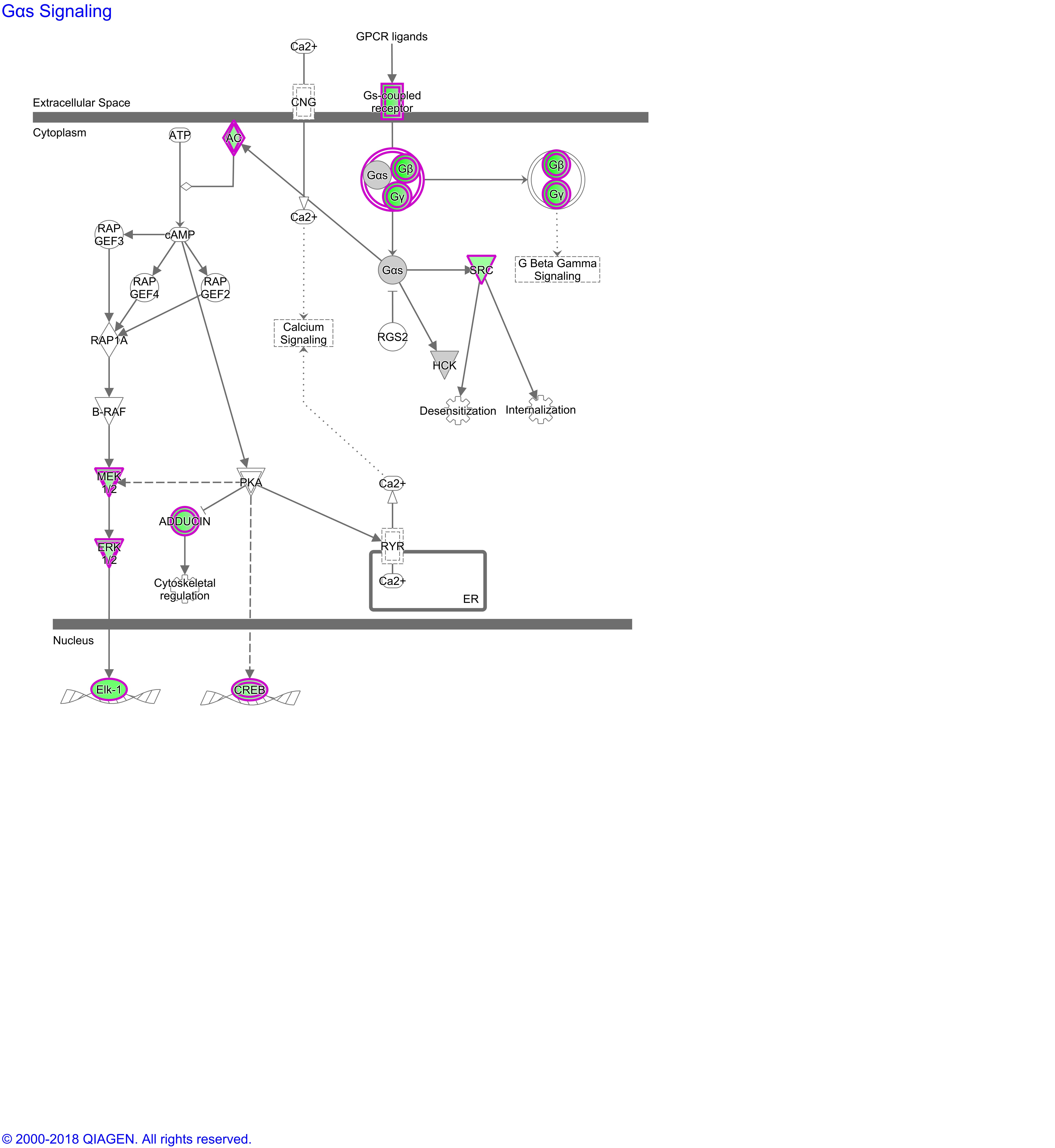

Supplement: Supplemental Figure 6 — Ingenuity pathway interaction network analysis of Gas signaling pathway. Differentially expressed genes between 20 week old dorsolateral prostate of TRAMP mice exhibiting alterations in elements of Gas signaling pathway compared to age-matched non-transgenic littermates. [file Image_6.JPEG]

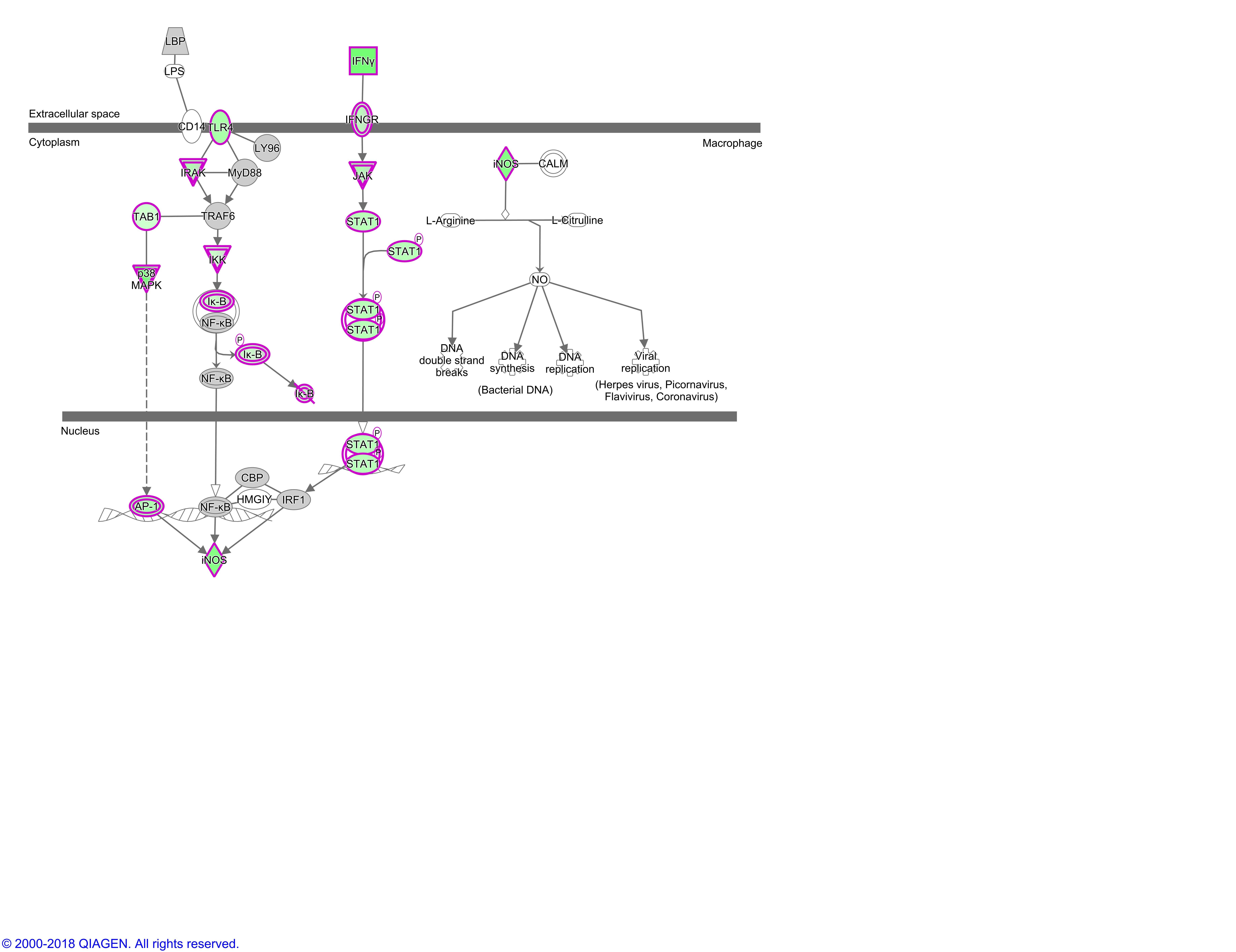

Supplement: Supplemental Figure 7 — Ingenuity pathway interaction network analysis of iNOS signaling pathway. Differentially expressed genes between 20 week old dorsolateral prostate of TRAMP mice exhibiting alterations in elements of iNOS signaling pathway compared to age-matched non-transgenic littermates. [file Image_7.JPEG]

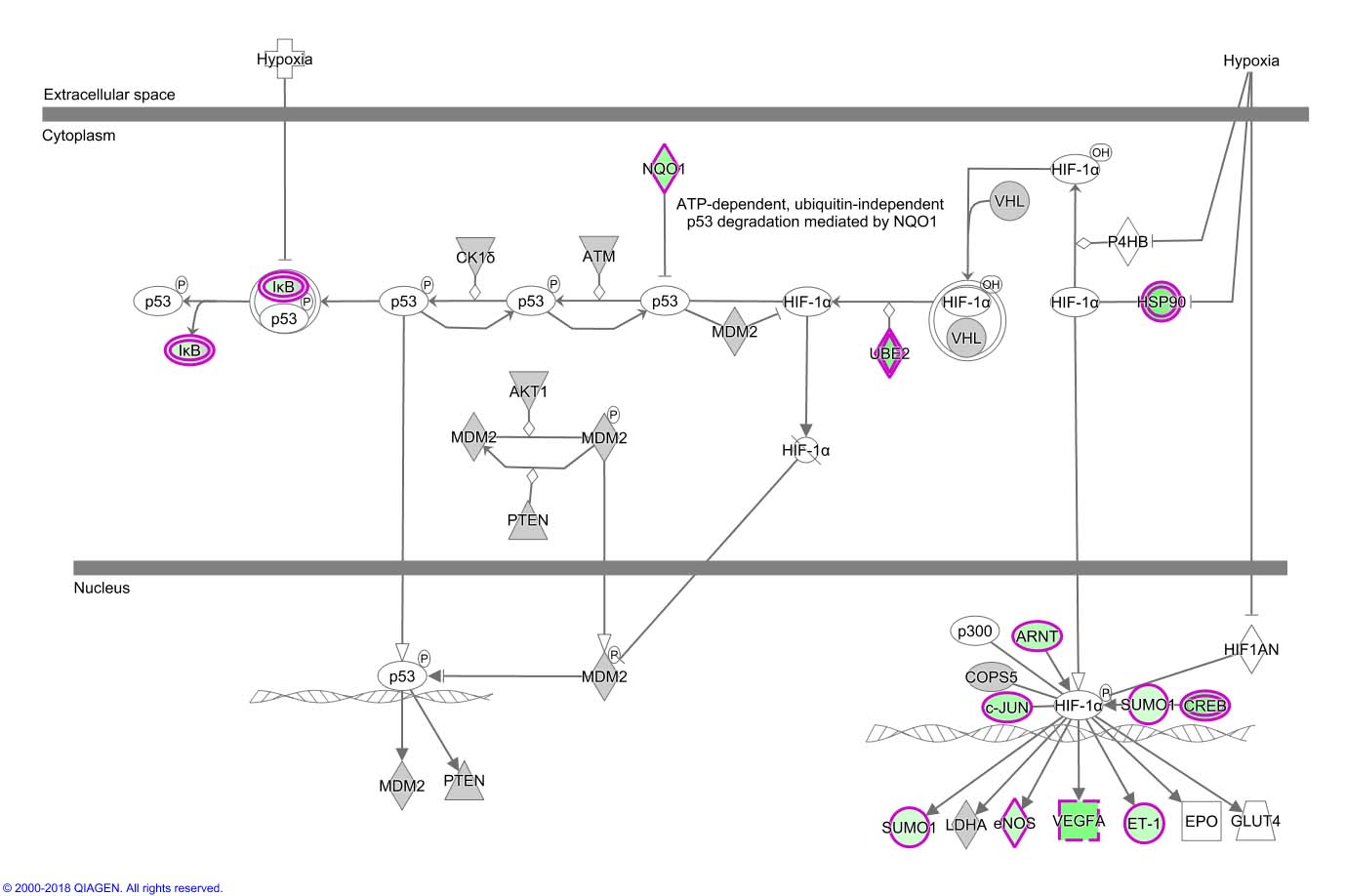

Supplement: Supplemental Figure 8 — Ingenuity pathway interaction network analysis of hypoxia signaling pathway. Differentially expressed genes between 20 week old dorsolateral prostate of TRAMP mice exhibiting alterations in elements of hypoxia signaling pathway compared to age-matched non-transgenic littermates. [file Image_8.JPEG]

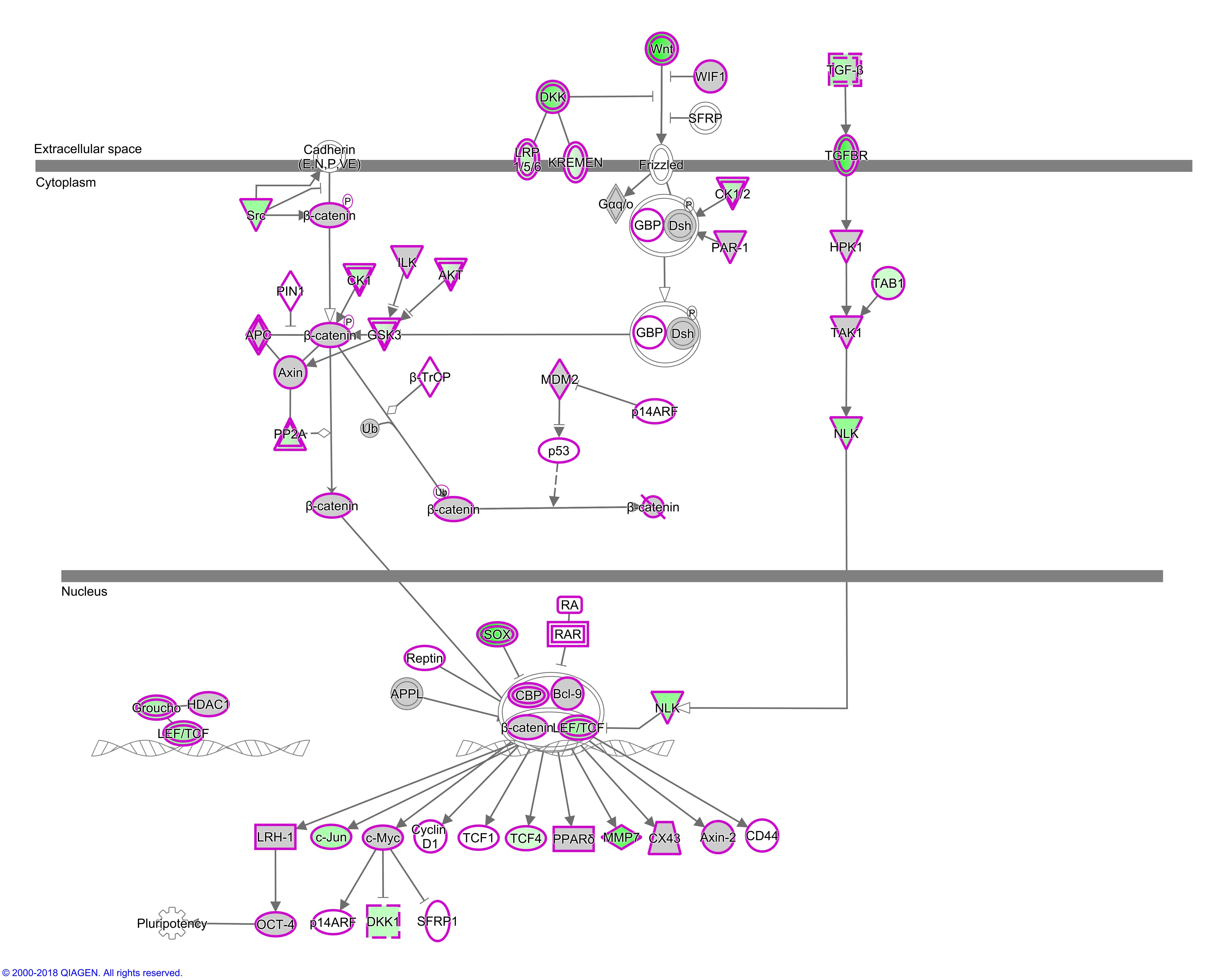

Supplement: Supplemental Figure 9 — Ingenuity pathway interaction network analysis of Wnt/β-catenin signaling pathway. Differentially expressed genes between 20 week old dorsolateral prostate of TRAMP mice exhibiting alterations in elements of Wnt/β-catenin signaling pathway compared to age-matched non-transgenic littermates. [file Image_9.JPEG]

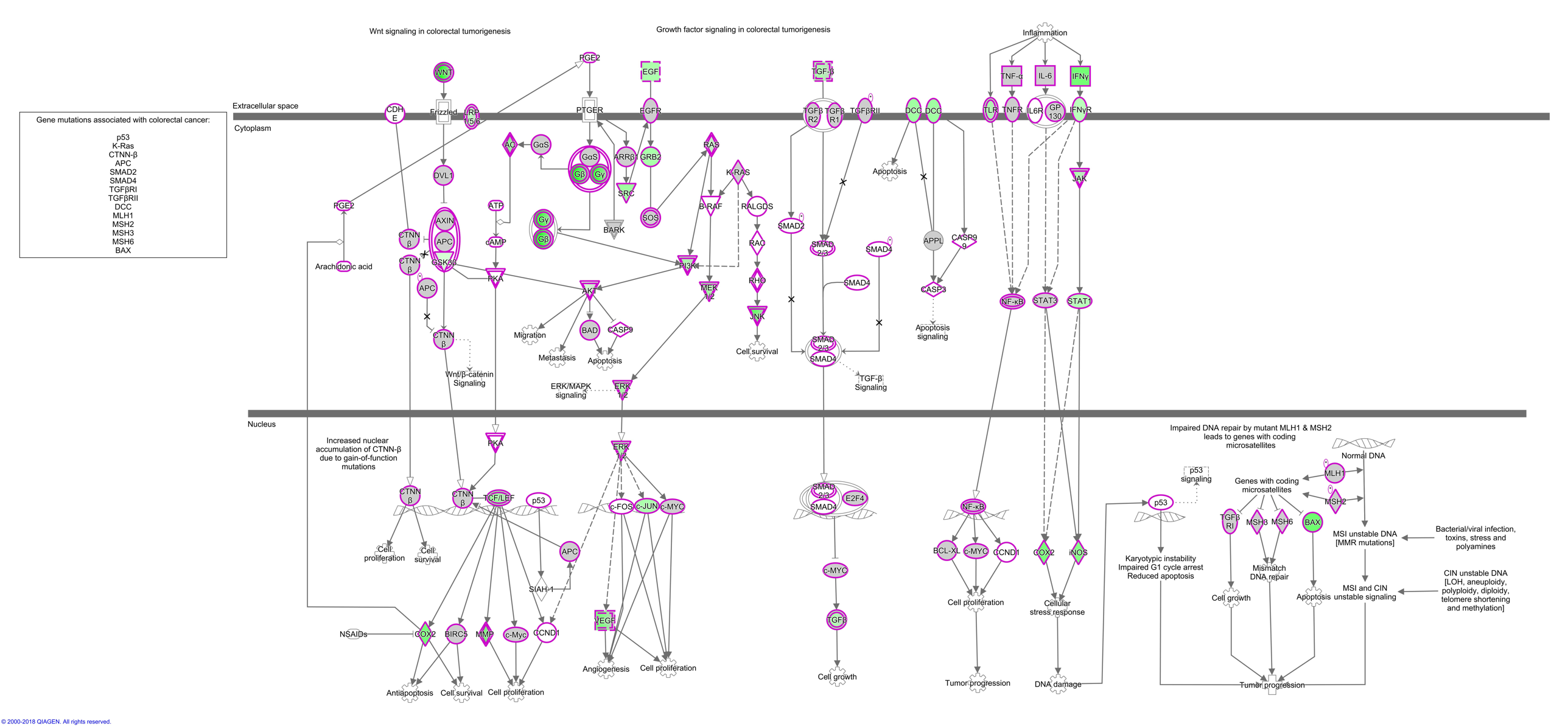

Supplement: Supplemental Figure 10 — Ingenuity pathway interaction network analysis of Cancer metastasis signaling pathway. Differentially expressed genes between 20 week old dorsolateral prostate of TRAMP mice exhibiting alterations in elements of metastasis signaling pathway compared to age-matched non-transgenic littermates. [file Image_10.JPEG]

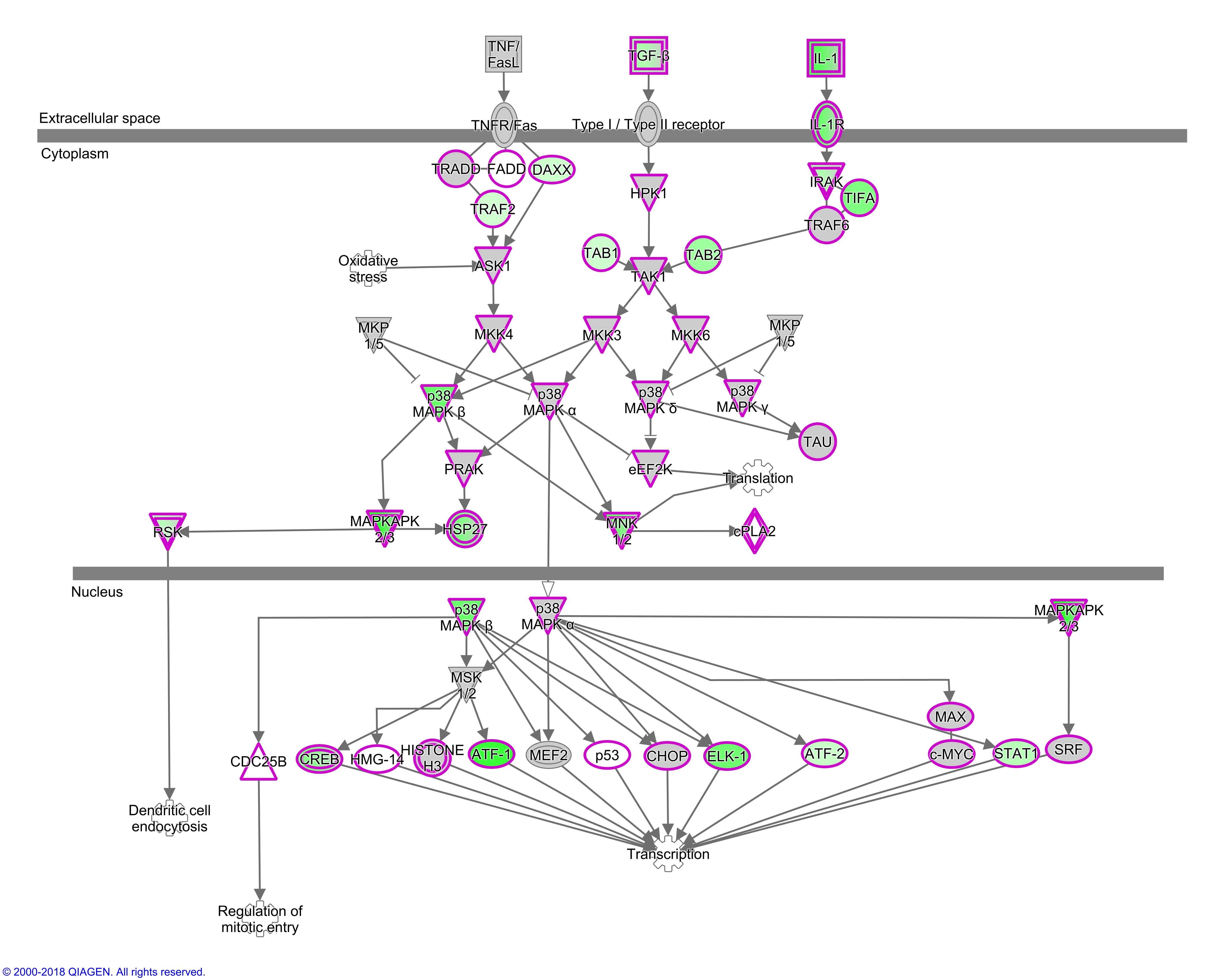

Supplement: Supplemental Figure 11 — Ingenuity pathway interaction network analysis of p38MAPKsignaling pathway. Differentially expressed genes between 20 week old dorsolateral prostate of TRAMP mice exhibiting alterations in elements of p38MAPKsignaling pathway compared to age-matched non-transgenic littermates. [file Image_11.JPEG]
