# Supplementary material for: Differentially Expressed Genes and Molecular Pathways in an Autochthonous Mouse Prostate Cancer Model
Source: Front Genet. 2019 Mar 26;10:235. doi: 10.3389/fgene.2019.00235 (PMC6445055; doi:10.3389/fgene.2019.00235)
Supplement: Supplemental Table 1 — List of set of primers of qRT-PCR with sequence (forward and reverse) used in the experiment to validate microarray data. [file Table_1.DOCX]

**Supplemental Table 1.** List of set of primers of qRT-PCR with sequence (forward and reverse) used in the experiment to validate microarray data.

| **S. No.** | **Gene** |  | **Primer Sequence (5'….3')** |
| --- | --- | --- | --- |
| 1 | Arfgap2 | Forward | CCAGCATCACTTACGGTGTAT |
| 1. | Arfgap2 | Reverse | CCAACTCTGTGGACCTGATAAA |
| 2 | Atp5l | Forward | AGACGTACCTTCCACCTTAGA |
| 2. | Atp5l | Reverse | CAATCGAGGCTTCGAGTAAGT |
| 3 | DIMT1 | Forward | CGCGTGGACCATCTAATGAA |
| 3. | DIMT1 | Reverse | GTGGCGGATTCTTAGGTTCTATC |
| 4 | GMCL1P1 | Forward | CTGGCACTGTCTTCTGACTTAC |
| 4. | GMCL1P1 | Reverse | CCGTTCCAGCATGGATTAAGA |
| 5 | Klf4 | Forward | CCCTTCGGTCATCAGTGTTAG |
| 5. | Klf4 | Reverse | GGACCGCCTCTTGCTTAAT |
| 6 | Mpo | Forward | GATCATCACATACCGGGACTAC |
| 6. | Mpo | Reverse | GGGTCTACTGAGTCGTTGTAAG |
| 7 | Mrpl34 | Forward | ACTGCACAGCCTGAAGATAC |
| 7. | Mrpl34 | Reverse | GCCACGGGAACATTGTAAATC |
| 8 | Naca | Forward | GAGGAACAAGACTCCACACAG |
| 8. | Naca | Reverse | TCGACTCTGCTTGGCTTTAC |
| 9 | Rpl22 | Forward | GTGCTCTCTCTGCAGGTATTT |
| 9. | Rpl22 | Reverse | CGCAGCTCGTAACTCTCTTT |
| 10 | SPRR1a | Forward | CTGAAGACCTGATCACCAGATG |
| 10. | SPRR1a | Reverse | GTGCAAGGAGAGAGGGATTAAG |
| 11 | Sostdc1 | Forward | CACAGTCCACAGTGCAGAA |
| 11. | Sostdc1 | Reverse | GGGAATGAGAGAGAGATGAATGG |
| 12 | Sp140 | Forward | CGGAGCAGAAGTTTCAGGAATA |
| 12. | Sp140 | Reverse | CTTTCAGAAGATCCCGGCTAAA |
| 13 | Tgm4 | Forward | CAGTCTGGCGTAGAGGTTATTC |
| 13. | Tgm4 | Reverse | CCACGGATTGAAGAGAAGGTAG |
| 14 | VDAC3 | Forward | GTTGGTTCGAGAAGACCTTCAG |
| 14. | VDAC3 | Reverse | GCCTTTCCTAGGTCGCAATAA |
